# Genome-wide characterisation and expression profile of the grapevine ATL ubiquitin ligase family reveal biotic and abiotic stress-responsive and development-related members

**DOI:** 10.1038/srep38260

**Published:** 2016-12-02

**Authors:** Pietro Ariani, Alice Regaiolo, Arianna Lovato, Alejandro Giorgetti, Andrea Porceddu, Salvatore Camiolo, Darren Wong, Simone Castellarin, Elodie Vandelle, Annalisa Polverari

**Affiliations:** 1 Dipartimento di Biotecnologie, Università degli Studi di Verona, Strada Le Grazie 15, Verona, 37134, Italy; 2 Università degli Studi di Sassari, Dipartimento di Agraria, SACEG, Via Enrico De Nicola 1, Sassari, 07100, Italy; 3 Wine Research Centre, University of British Columbia, 326–2205 East Mall, Vancouver, BC V6T 1Z4, Canada

## Abstract

The Arabidopsis Tóxicos en Levadura (ATL) protein family is a class of E3 ubiquitin ligases with a characteristic RING-H2 Zn-finger structure that mediates diverse physiological processes and stress responses in plants. We carried out a genome-wide survey of grapevine (*Vitis vinifera* L.) ATL genes and retrieved 96 sequences containing the canonical ATL RING-H2 domain. We analysed their genomic organisation, gene structure and evolution, protein domains and phylogenetic relationships. Clustering revealed several clades, as already reported in *Arabidopsis thaliana* and rice (*Oryza sativa*), with an expanded subgroup of grapevine-specific genes. Most of the grapevine ATL genes lacked introns and were scattered among the 19 chromosomes, with a high level of duplication retention. Expression profiling revealed that some ATL genes are expressed specifically during early or late development and may participate in the juvenile to mature plant transition, whereas others may play a role in pathogen and/or abiotic stress responses, making them key candidates for further functional analysis. Our data offer the first genome-wide overview and annotation of the grapevine ATL family, and provide a basis for investigating the roles of specific family members in grapevine physiology and stress responses, as well as potential biotechnological applications.

The selective degradation of ubiquitinylated proteins by the 26*S* proteasome is a key mechanism controlling a variety of processes in eukaryotic cells, particularly developmental and stress-related signalling^1^,^2^. Ubiquitin is covalently attached to the lysine residues of target proteins via an ATP-dependent reaction cascade that involves the sequential action of three enzymes: E1 (ubiquitin activating), E2 (ubiquitin conjugating) and E3 (ubiquitin ligase)^3^,^4^. The protein substrate can undergo monoubiquitinylation or polyubiquitinylation, the latter resulting in proteolytic degradation by the 26*S* proteasome and the former triggering protein translocation often followed by lysosomal degradation^5^.

E3 ubiquitin ligases are by far the most diverse and abundant enzymes in the ubiquitinylation cascade in plants, reflecting their versatile activity in different pathways leading to proteolytic degradation^6^. These pathways are active not only under physiological conditions, but also in response to stress and particularly during plant–pathogen interactions^4^,^7^.

There are four classes of E3 ubiquitin ligases based on conserved structural features and mechanisms of action^8^. One class is defined by the REALLY INTERESTING NEW GENE (RING) domain first identified in the human RING1 gene, comprising eight precisely spaced cysteine and histidine residues that coordinate two Zn ions^9^. RING-containing proteins are important because they are abundant among the E3 ligase classes in plants and can ubiquitinylate substrates independently or participate in multi-subunit complexes, thus regulating a number of different physiological responses. Moreover, the RING domain is responsible for binding to E2 conjugating enzyme within the ubiquitinylation cascade^4^,^10^. The Arabidopsis Tóxicos en Levadura (ATL) protein family features a specific variant of the RING domain (RING-H2) and is so named because the first member (AtATL2) identified in Arabidopsis (*Arabidopsis thaliana*) showed conditional toxicity when overexpressed in yeast^11^. In the ATL RING-H2 domain, the fourth and fifth Zn-coordinating ligands are histidine residues, and a proline residue is found adjacent to the third cysteine residue, resulting in the consensus Cys-X(2)-Cys-X(n)-Px-Cys-X(1)-His-X(2)-His-X(2)-Cys-X(n)-Cys-X(2)-Cys. Other features conserved in most known ATL proteins are a tryptophan residue located three residues downstream from the forth cysteine residue, an N-terminal hydrophobic region, a GLD motif, and a basic region, but these modules are not always present and therefore are not considered a requirement for ATL classification^12^. A variant of the ATL protein structure was recently described in Arabidopsis in which an additional BCA2 Zinc Finger (BZF) domain replaces the typical hydrophobic domain at the N-terminus^13^. The BZF domain, first identified in the N-terminal region of the human BCA2 RING E3 ligase^14^, interacts with ubiquitin and is required for E3 ligase activity^15^.

The putative plant orthologues of RING-H2 finger proteins that contain a BZF domain are known as BTL proteins (BZF ATLs). In these proteins, the proline residue associated with the RING-H2 domain is immediately adjacent to the third cysteine residue (P-Cys instead of P-X-Cys). Seventeen such BTL proteins have been described in Arabidopsis thus far^13^.

ATL proteins in different plant species have attracted much recent attention because they may help plants to adapt to environmental stress, possibly through ubiquitin-mediated protein degradation^16^. Several ATL proteins are rapidly and transiently induced in response to elicitors and may therefore mediate defence responses, including Arabidopsis ATL2 and ATL6, rice (*Oryza sativa*) EL5^17^,^18^, and tomato (*Solanum lycopersicum*) LeATL6, which is probably involved in jasmonic acid signalling^19^. The phenotypes of plants in which ATL genes have been overexpressed or silenced, and also those of mutants with altered ATL expression levels, suggest some ATL proteins play a role in defence responses, including Arabidopsis ATL2^20^ and ATL9^21^, potato (*Solanum tuberosum*) StRFP1, and rice BIRF1^22^,^23^,^24^. In some cases, the more robust defence responses achieved by higher ATL expression was accompanied by negative pleiotropic effects on development^20^,^24^, in agreement with the common observation of an antagonistic correlation between resistance and developmental events^25^. Other experiments reveal that ATL proteins have a direct functional role in abiotic stress responses, especially those mediated by abscisic acid (ABA). These include the Arabidopsis proteins ATL43 and ATL78, which respectively control ABA sensitivity during germination^12^ and ABA-mediated responses to drought stress^26^, as well as the soybean (Glycine max) ATL protein GmRFP1, which is also involved in ABA signalling and stress responses^27^. Finally, several ATL proteins can also regulate plant metabolism, development and flowering, such as Arabidopsis ATL25, ATL32 and ATL62 (previously identified as the factor DAY NEUTRAL FLOWERING or DNF), and rice EL5^28^,^29^,^30^,^31^. Sometimes these proteins have interconnected roles, as reported for Arabidopsis ATL80, which was shown to negatively affect both phosphorus mobilisation and the cold stress response^32^. In most cases, the ubiquitinylation target of the individual ATL proteins is unknown.

The publication of the grapevine (*Vitis vinifera*) genome sequence in 2007 facilitated the functional analysis of a number of gene families characterised in other species to determine whether these could be used to improve the performance of this economically important fruit crop^33^,^34^,^35^,^36^,^37^,^38^,^39^. The E3 ubiquitin ligase family has not been investigated thus far, and only one grapevine RING-type E3 ubiquitin ligase has been functionally characterised: the EIRP1 protein from the wild species *V. pseudoreticulata*. In yeast two-hybrid experiments, recombinant EIRP1 was shown to interact with the defence-related transcription factor VpWRKY11 and to regulate its activity via a proteasome-dependent mechanism^40^.

We have previously investigated the general and species-dependent transcriptional responses of the susceptible grapevine species *V. vinifera* and its resistant wild North American relative *V. riparia* to *Plasmopara viticola*, the agent responsible for downy mildew^41^. This revealed a group of eight RING-H2 genes with the typical ATL signature that were strongly upregulated in response to the pathogen specifically in the resistant species *V. riparia*. To investigate this interesting gene family in more detail, we therefore carried out a whole-genome characterisation as an essential starting point for the functional analysis of ATL proteins that could potentially be exploited in the future to introduce disease resistance into today’s susceptible grapevine cultivars.

## Results

### Genome-wide identification and annotation of grapevine ATL genes

We surveyed the grapevine genome (*V. vinifera* cv Pinot Noir, genotype PN40024) to identify all genes containing a canonical ATL-type RING-H2 domain^13^,^42^. Each potential RING-H2 domain was analysed for the presence of, and the distance between, each of the Zn-coordinating cysteine and histidine residues, as well as the presence of a proline residue before the third cysteine according to the original ATL definition reported for the ATL family in Arabidopsis^12^.

**Figure 1:**
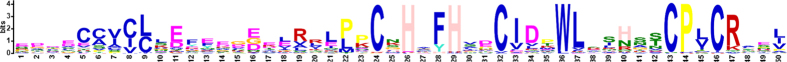
Sequence LOGO of the VviATL RING-H2 domains. The LOGO was generated from the protein sequences of the 96 ATLs identified in the whole *Vitis vinifera* genome.

The presence of a particular N-terminal domain and the spacing between proline and cysteine were not considered to be discriminating criteria because the careful visual inspection of grapevine proteins containing an ATL RING-H2 domain, and the exploration of literature and database resources, revealed a complex scenario in which there was no clear distinction between ATL and BZF-containing ATL proteins. Among the 96 identified ATLs (Table 1 and Supplemental Fig. 1), 45 contained an N-terminal transmembrane domain or at least a hydrophobic region and the PxC motif in the RING-H2 domain, 10 contained an N-terminal BZF domain together with a PC motif in the RING-H2 domain, 25 proteins displayed the N-terminal hydrophobic domain typical of ATLs, despite a BTL-like PC motif near the RING-H2 domain, 3 proteins contained both the BZF domain and an hydrophobic domain at the N-terminal together with a PC signature, and 16 proteins did not contain any particular N-terminal domain other than the PC or PxC motif. All of the selected proteins also contained a tryptophan residue three places downstream from the forth cysteine residue. The LOGO diagram of the RING-H2 domain for the 96 grapevine ATLs is shown in Fig. 1 and the alignment of the RING-H2 domain amino acid sequences is shown in Supplemental Fig. 2.

The resulting list of 96 ATL genes was visually curated to ensure that each gene was expressed in grapevine by checking published whole-genome microarray and RNAseq experiments^41^,^43^. All 96 ATLs were expressed above the background level of detection as defined in these earlier studies.

### Phylogenetic analysis and nomenclature of the grapevine ATL genes

Phylogenetic analysis of the nucleotide sequences of the 96 grapevine ATL genes and the 83 Arabidopsis ATL genes was carried out to determine the evolutionary relationships among the genes. The aim was to assign a name to each grapevine ATL gene according to the guidelines and statistical tools defined by the international Super-Nomenclature Committee for Grape Gene Annotation (sNCGGa)^44^.

**Figure 2:**
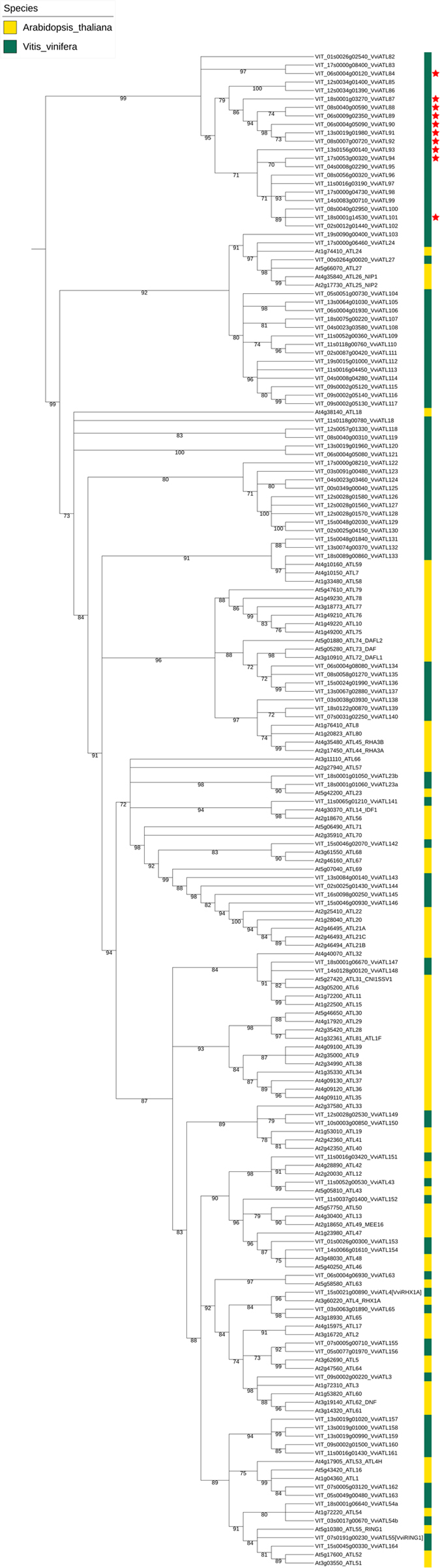
Phylogenetic analysis of the *V. vinifera and A. thaliana* ATL genes. The unrooted tree was generated with the Phylogeny.fr suite (http://www.phylogeny.fr) using the full-length nucleotide coding sequences of the 96 grapevine ATL genes identified herein (in green) and the 83 ATL genes of *A. thaliana* reported in the UniProt database (in yellow). Branch support values were obtained from 100 bootstrap replicates. The red stars indicate the presence of a BZF domain in the corresponding proteins.

Following this approach, 13 of the 96 grapevine ATL gene nucleotide sequences paired with orthologues in the Arabidopsis genome (Fig. 2). The phylogenetic tree revealed that grapevine ATL genes are dispersed across the dendrogram but tend to cluster in a species-dependent manner. For example, one cluster contains 55 grapevine ATL genes but only four Arabidopsis genes, suggesting this subgroup (which includes the 10 BTL-type ATL genes) expanded after the separation of Arabidopsis and grapevine from their common ancestor. Although most genes in this cluster possess a BTL-type RING-H2 signature, many do not contain a BZF domain and some have one or multiple transmembrane regions. Based on this tree, the grapevine ATL genes were named according to the abovementioned guidelines^44^. When one-to-one orthologues were identified, the grapevine gene was given the same name as its counterpart in Arabidopsis (e.g. VviATL43 is the orthologue of AtATL43). Otherwise, the grapevine genes were assigned a functional name (ATL) followed by a number higher than the highest number used for Arabidopsis. Therefore, the progressive numbering of grapevine gene names proceeds along the phylogenetic tree (Table 1). If two or more grapevine genes are placed at the same phylogenetic distance from a single Arabidopsis gene, they were differentiated by a letter (e.g. VviATL23a and VviATL23b are both homologous to AtATL23). When one or more genes in grapevine matched more than one gene in Arabidopsis, a new name was attributed consisting of the common ATL term as the root and the next independent number as an index (e.g. VviATL154 matches both AtATL46 and AtATL48). The Locus ID from the V1 grapevine genome browser (http://genomes.cribi.unipd.it/grape/)^45^ is also shown in Fig. 2 and Table 1 to provide a unique identifier and to avoid mistakes during future expansion of the nomenclature or conversion from different sources.

### Chromosomal location, duplications and exon–intron organisation

**Figure 3:**
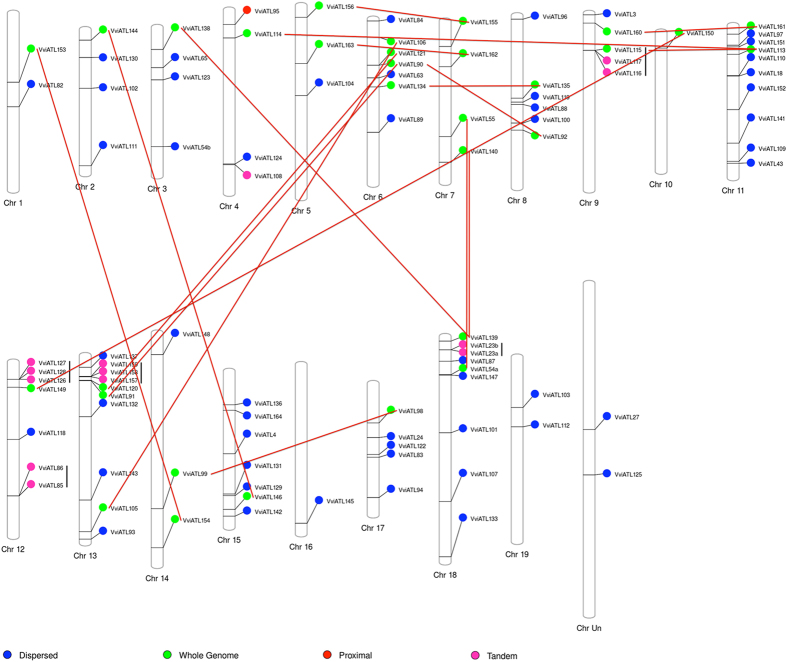
Distribution of grapevine ATL gene family members among the *Vitis vinifera* chromosomes. The 96 grapevine ATL genes with exact chromosomal information available in the database were mapped to the 19 *V. vinifera* chromosomes. The colours indicate the original duplication event. Vertical black lines and red lines identify pairs derived from tandem duplications and whole genome duplications, respectively.

**Figure 4:**
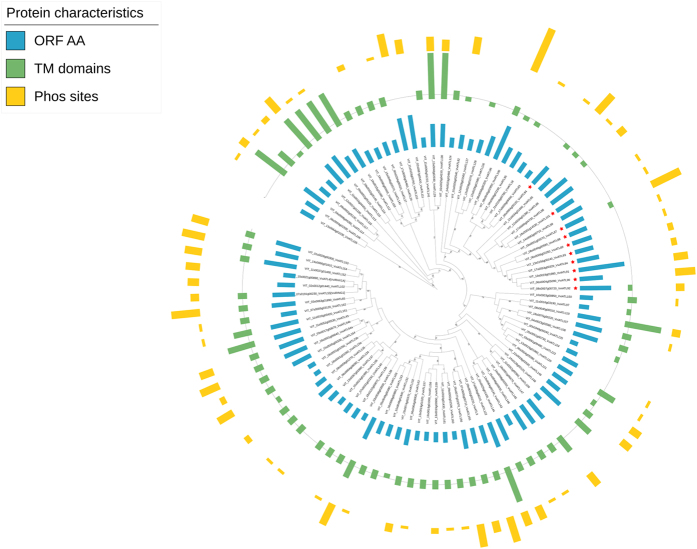
Phylogenetic analysis and main features of grapevine ATL proteins. The unrooted tree was generated with the Phylogeny.fr suite (http://www.phylogeny.fr) using the full-length protein sequences of the 96 grapevine ATLs identified herein. Branch support values were obtained from 100 bootstrap replicates. For each ATL protein, the protein length (blue bars), the presence of transmembrane/hydrophobic domains (in green) and putative phosphorylation sites predicted with MUsite v1.0 (in yellow) are shown. The length of coloured bars is proportional to the number of amino acids, TM or hydrophobic domains and phosphosites, respectively. The grey line represents the threshold for a domain to be considered as a TM (above the line) or hydrophobic domain (on the line). Red stars represent the presence of a BZF domain in the corresponding protein.

We mapped 94 of the 96 ATL sequences to the 19 grapevine chromosomes, and the other two were assigned to the unknown chromosome (chrUn) as shown in Table 1 and Fig. 3. The ATL genes were distributed throughout the genome, indicating that whole-genome duplication was an important evolutionary mechanism underlying the expansion of the grapevine ATL gene family. We also found couples or clusters of adjacent homologs reflecting tandem duplication events (e.g. on chromosomes 9, 12, 13 and 18). Accordingly, 31 ATL genes were found in homologous chromosomal regions derived from segmental or whole genome duplications, 13 from tandem duplications, 1 from a proximal duplication and 51 from dispersed duplications (Fig. 4). Notably, the paralogue of the tandemly-duplicated *VviATL108* gene (VIT_ 04s0023g03580) does not belong to the ATL family because the corresponding protein does not contain the RING-H2 domain required for ATL classification in this study. The presence of several ATL clusters caused by duplication events resulted in a heterogeneous distribution of ATL genes among the chromosomes, with chromosomes 11, 13 and 18 containing the highest number of ATL genes and the presence of four tandem duplication clusters on chromosomes 9, 12, 13 and 18. Enrichment tests indicated that the segmental/whole-genome duplication ATL members were preferentially retained during genome fractionation (*p* < 0.001, Fisher’s exact test) suggesting that the ATL family played a role in grapevine adaptation and evolution.

As in other species, the grapevine ATL family has a moderate to low complexity in terms of gene structure, with ~50% the genes containing no introns and ~78% containing from zero to three introns. Interestingly, amino acid sequence similarity within the RING-H2 domain was reflected to some extent by the complexity of gene structure (Supplemental Fig. 3), with the lower part of the phylogenetic tree formed mostly by intronless genes and the number of introns tending to increase towards the top of the tree. Detailed information about the identified grapevine ATL genes/proteins is provided in Table 1 and Supplemental Table 1, including nomenclature, accession numbers, chromosomal locations, gene and protein lengths, and predictions of the isoelectric point (pI), molecular weight (MW), subcellular localisation and phosphorylation sites.

### Additional features of the grapevine ATL proteins

In addition to the typical RING-H2 domain, 17 of the putative ATL proteins also contained a complete GLD motif, 23 contained two of the conserved residues (GLx or GxD) and 12 only the conserved glycine residue (Gxx) in the appropriate position (Supplemental Fig. 4). Only 19 further domains in 14 sequences were detected in addition to the common RING-H2 sequence (Table 1). Ten of the ATL proteins contained an N-terminal BZF domain (C2/C2 zf-RING_3: PF14369)^15^ as described for the BTL subfamily in Arabidopsis^13^. We also identified three domain-of-unknown-function (DUF) 1117 motifs, also present, among others, in two Arabidopsis RING domain-containing E3 ubiquitin ligases (*AtRDUF1* and *AtRDUF2*)^46^, and two protease-associated domains (PF02225), which could act as a lid covering the remnants of the active site of catalytically inactive proteins^47^. The other domains we identified were present in individual proteins: a wall-associated receptor kinase galacturonan-binding (GUB_WAK_bind) domain (PF13947), a cysteine-rich, pectin-binding domain found in cell wall-associated serine/threonine kinases (WAKs)^48^, a Rhodanese-like domain (PF00581) found in enzymes involved in the detoxification of cyanide^49^, an Asp domain (PF00026) containing two conserved Asp residues as typically found in aspartic proteases, and a WAC_associated domain (PF14380), which is a wall-associated C-terminal receptor kinase often coupled to the GUB_WAK_bind domain mentioned above. As already stated, 58 of the grapevine ATL proteins contained up to five putative transmembrane domains and 15 contained at least one hydrophobic region (Table 1 and Supplemental Fig. 5).

TargetP, ngLOC and PProwler were used to predict the subcellular location of each grapevine ATL. Several ATLs were predicted to localise in the secretory pathway (i.e. presence of a signal peptide), whereas others were predicted to localise in the plasma membrane (in accordance with the transmembrane domain structure), but also in plastids, mitochondria, the endoplasmic reticulum or nucleus (Supplemental Table 1). The predicted ATL localisation did not appear to depend on the nature of the N-terminal domains or RING-H2 motif.

Given that ATL protein activity can be regulated by phosphorylation^50^,^51^, we also evaluated the presence of putative phosphorylation sites using Musite. We thus identified at least one putative phosphorylation site in 69 of the 96 candidates, with a maximum of 16 putative phosphorylation sites identified in VviATL100 (Supplemental Table 1). The putative phosphorylation sites were mostly serine residues.

To provide a comprehensive view of grapevine ATL protein relationships, a phylogenetic tree is presented in Fig. 4, which also reports the relevant features of the proteins, such as their length, the presence of transmembrane domains or hydrophobic regions and the predicted number of phosphorylation sites.

### The spatiotemporal expression profile of grapevine ATL genes

**Figure 5:**
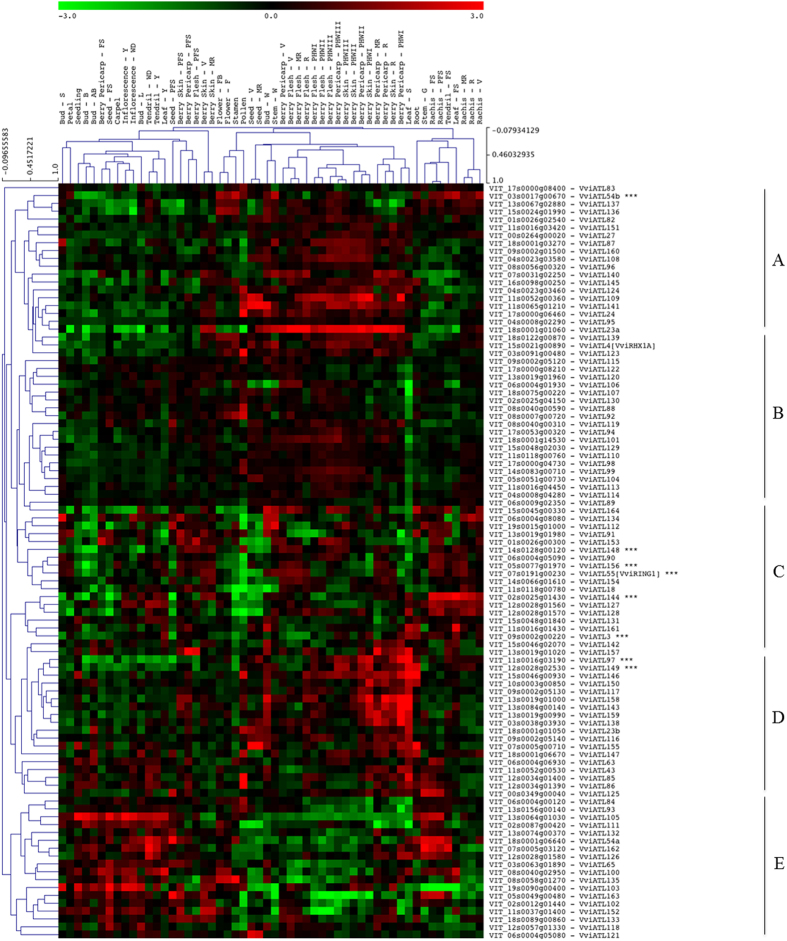
Hierarchical clustering of the expression profiles of grapevine ATL genes in different organs. The log transformed expression values of grapevine ATL genes in the grapevine atlas^43^ were used for hierarchical cluster analysis based on Pearson’s distance metric. The colour scale represents higher (red) or lower (green) expression levels with respect to the median transcript abundance of each gene across all samples. Letters A to E on the right side indicate the different clusters identified. AB: after burst; B: burst; bud-W: winter bud; F: flowering; FB: flowering begins; FS: fruit set; G: green; MR: mid-ripening; PFS: post-fruit set; PHWI-II-III: post-harvest withering 1, 2 and 3 months; R: ripening; S: senescent; stem-W: woody stem; V: veraison; WD: well developed; Y: young.

To biological function of the grapevine ATL genes was investigated by retrieving their expression profiles from the *V. vinifera* cv. Corvina global gene expression atlas, a whole-genome expression survey of 54 different grapevine organs and tissues at various developmental stages, obtained by NimbleGen microarray analysis^43^. All 96 ATL transcripts were represented by probes on the NimbleGen array, and the fluorescence intensity values were used to generate a biclustered heat map in which the data were normalised based on the mean centre genes/rows adjustment method (Fig. 5). All 96 ATL genes were expressed in at least one of the 54 tissues/stages. Overall we could distinguish five main groups based on the clustering of expression profiles. Interestingly, clusters A and E showed opposing upregulation or downregulation in some groups of organs and stages. Cluster A genes were typically downregulated in juvenile samples, including early berry stages, young leaf, tendril and inflorescence, and most of the bud stages, but were upregulated in mature samples, including berry tissues at the ripening and post-harvest withering stages, woody buds and stems, and late stages of seed development, whereas cluster E genes showed broadly the opposite behaviour. Cluster C included genes that tended to be downregulated in most of the samples (developmental stages and organs) except rachis and tendrils in which expression increased, whereas genes belonging to cluster D tended to be mostly upregulated only in the late phases of development, mainly in berries. Finally, cluster B comprised genes that did not show any particular variation. There was a very low correlation between the phylogenetic tree and the expression profile clusters during grapevine organ development, even for duplicated genes (Figs 3 and 5). Statistical analysis revealed only a small positive association (Monte-Carlo test, observation = 0.08, simulated p-value = 0.002) between ATL sequence similarity and ATL expression patterns in grapevine organs, suggesting that the ATL family has undergone sub-functionalisation after duplication.

### Expression of grapevine ATL genes in response to biotic stress

**Figure 6:**
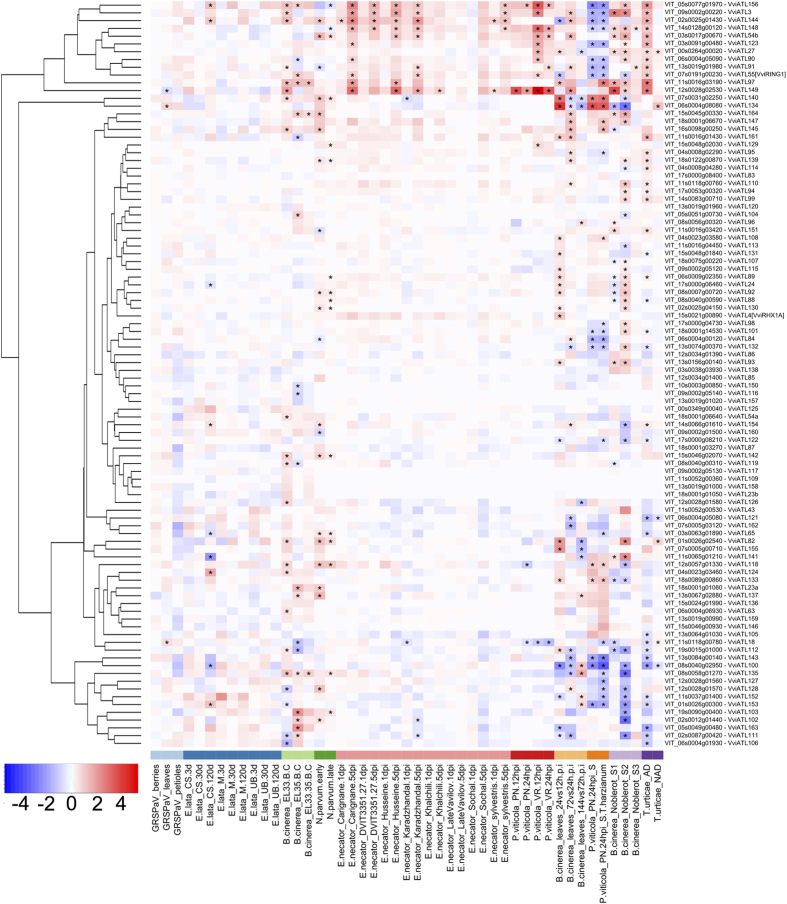
Hierarchical clustering of the expression profiles of grapevine ATL genes in the grapevine–pathogen interaction dataset. The colour scale represents increased (red) or decreased (blue) fold changes of grapevine ATL gene expression in infected samples compared to controls for each condition. Asterisks indicate the significant differential expression (FDR < 0.5) of each ATL under the corresponding conditions. References to the published datasets and differential expression criteria are reported in the Materials and Methods.

Further insight into the potential functions of specific ATL proteins was gained by investigating a condition-specific transcriptomic dataset based on microarray and RNA-seq experiments involving biotic stress, available in the Gene Expression Omnibus (GEO) and ArrayExpress databases. The conditions included infections with different viral and fungal pathogens as well as herbivorous parasites attacking grapevine leaves, stems, trunks or berries (Fig. 6 and Supplemental Table 2). Some experimental datasets (identified by light and dark green, light and dark orange and light and dark purple bars in Fig. 6) showed a larger number of differentially expressed ATL genes, in most cases probably due to the greater sensitivity of the RNA-seq technique. Altogether, 62 ATL transcripts showed evidence of significant modulation with a log2 fold-change (FC) >|0.5| under at least two conditions, with a false discovery rate (FDR) < 0.05. This increased to 81 ATL transcripts if significant modulations under at least one condition were included regardless of the FC value, suggesting that grapevine ATL genes are generally responsive to pathogens. Moreover, the clustering of the FC values (Fig. 6) clearly revealed a group of 12 ATL genes: ATL3, ATL27, ATL54b, ATL55, ATL90, ATL91, ATL97, ATL123, ATL144, ATL148, ATL149 and ATL156 (VIT_09s0002g00220, VIT_00s0264g00020, VIT_03s0017g00670, VIT_07s0191g00230, VIT_06s0004g05090, VIT_13s0019g01980, VIT_11s0016g03190, VIT_03s0091g00480, VIT_02s0025g01430, VIT_14s0128g00120, VIT_12s0028g02530, VIT_05s0077g01970). These were strongly upregulated in response to most pathogens, including biotrophic fungi (*Erysiphe necator* and *Plasmopara viticola*), necrotrophic fungi (*Botrytis cinerea* and *Neofusicoccum parvum*) and herbivores (*Tetranychus urticae*). In response to powdery mildew, these genes were upregulated in several resistant accessions as well as a susceptible one, and in response to *B. cinerea* they were upregulated regardless of the outcome of the infection (grey mould or noble rot). Interestingly, infection with *P. viticola* caused the induction of nine of these 12 genes in the resistant species *V. riparia*, but they were predominantly downregulated in the susceptible species *V. vinifera*, along with most of the 62 differentially expressed ATL genes. This suggests that there could be a correlation between the suppression of ATL gene expression and susceptibility to *P. viticola*. Similarly, ATL genes that respond strongly to biotic stress were all upregulated in response to an adapted spider mite, but not in plants challenged with the non-adapted pest strain, supporting their putative role in specific defence responses. In contrast, ATLs are often upregulated when *B. cinerea* causes grey mould but not during the development of noble rot. Viral infection during the compatible interaction with *V. vinifera* did not modulate ATL gene expression with the exception of a slight and statistically insignificant downregulation of some ATL genes in the petioles of infected plants. Overall, these data support the involvement of the ATL gene family in a general response to biotic stress, not specific to necrotrophic or biotrophic pathogens, and highlight a group of ATL genes that respond more strongly to particular types of pathogens.

### Expression of grapevine ATL genes in response to abiotic stress

A similar bioinformatics survey was carried out to investigate the changes in ATL gene expression in response to different forms of abiotic stress, analysing the effects of dehydration, exogenous glucose treatment, heat stress and UV damage on grapevine berries, and the effect of carbon starvation on flowers obtained by shading grapevine plants at bloom. We found that 54 ATL transcripts were significantly modulated with a log2 FC value >|0.5| under at least two conditions, with a false discovery rate (FDR) < 0.05. This increased to 73 ATL transcripts significantly affected under at least one condition regardless of the FC value, indicating the general responsiveness of most grapevine ATL genes to abiotic stress.

The clustering of FC values revealed groups of ATL transcripts responding more intensely to stress, such as in clusters A, C and E. However, even within these clusters, the individual ATL family members responded differently to different forms of stress, both in quantitative terms and in the direction of change, so there was no uniform response to abiotic stress in general.

**Figure 7:**
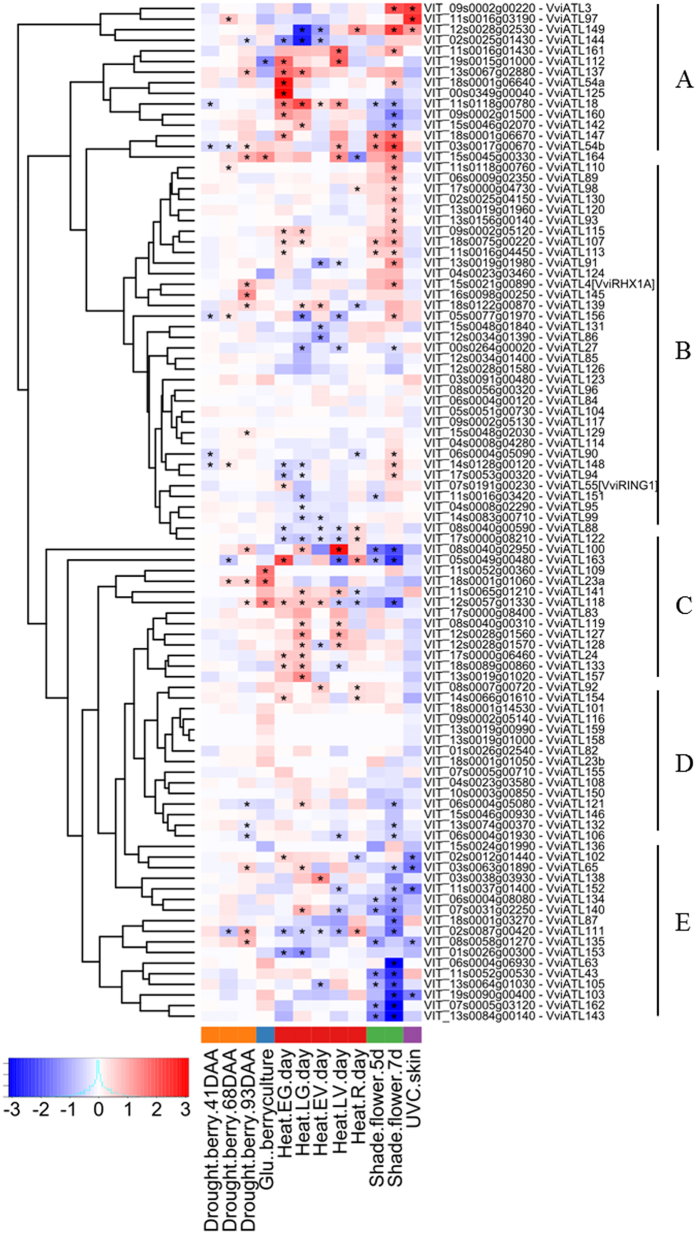
Hierarchical clustering of the expression profiles of grapevine ATL genes in the grapevine abiotic stress datasets. The colour scale represents increased (red) or decreased (blue) fold changes of grapevine ATL gene expression in samples exposed to abiotic stress compared to controls for each condition. Asterisks indicate the significant differential expression (FDR < 0.5) of each ATL under the corresponding conditions. References to the published datasets and differential expression criteria are reported in the Materials and Methods.

A wider effect was observed on inflorescences following carbon starvation (shading), in particular after 7 days, with 22 upregulated and 22 downregulated ATL transcripts (Supplemental Table 3 and Fig. 7). Heat stress also modulated a diverse set of berry ATL transcripts, mostly in the opposite direction to the effect of shading. Other forms of stress affected a lower number of berry ATL transcripts, including water deficit (26 genes), sugar treatment (5 genes) and UV damage (8 genes).

When comparing the list of the 54 stress-responsive ATLs with the list of 62 ATL transcripts modulated by pathogens and pests, we identified 41 overlapping genes that may be involved in a more general stress response (Supplementary Table 4 and Supplementary Figure 6).

## Discussion

The ubiquitinylation system is an important regulatory mechanism for a broad range of physiological and developmental processes in plants^4^. It also mediates abiotic and biotic stress responses, including the regulation of pathogen perception, downstream signal transduction cascades and hormone-related responses^7^,^52^. The importance of E3 ubiquitin ligases in the establishment of an immune response in plants is also highlighted by the fact that pathogen effectors often interfere with the proteasome system as a virulence strategy^4^,^53^,^54^. The plant-specific ATL family of E3 ubiquitin ligases is often involved in stress responses^42^. Following the observation that several ATL transcripts are strongly upregulated by *P. viticola* specifically in the resistant grapevine species *Vitis riparia*^41^, we carried out a genome-wide characterisation of grapevine ATL genes and explored their expression profiles under normal conditions and in response to biotic and abiotic stresses in a number of publicly available transcriptomic datasets. This will help to identify candidate genes involved in development and defence that could be used in the future to improve the agronomic properties of grapevine crops.

The survey of the grapevine genome retrieved 96 ATL genes, consistent with the ATL families described in Arabidopsis and rice^12^, but higher than the 30 ATL genes and the seven BTL genes identified in the first draft (V0, 8X prediction) grapevine genome sequence^13^. Among the 96 ATL proteins we identified, 10 contain a proline residue before the third cysteine residue of the canonical RING-H2 domain and therefore belong to the BTL subfamily. Additional features related to the N-terminal domain of the ATL proteins were not required as further criteria for inclusion in the family because these were not present in all proteins and it was more appropriate to consider the biologically active part of the protein (the RING-H2 domain) which is needed to bind E2 ubiquitin-conjugating enzymes^10^. Even so, we noted that 73 of the 96 ATL proteins contained at least one hydrophobic region, which may facilitate cellular targeting and responses to different signalling cues^42^. A few additional domains were identified in a small number of ATL proteins but most contained solely the RING-H2 domain, as reported in Arabidopsis^16^.

Phylogenetic analysis of the ATL nucleotide sequences from grapevine and Arabidopsis allowed the application of a systematic nomenclature^44^. The ATL sequences were diverse, so we used the Gblocks program with relaxed parameters to achieve sufficient numbers of aligned blocks to build the tree. This procedure was validated by the close proximity of paralogues and orthologues in the tree. The grapevine genes were dispersed across the dendrogram but often clustered in small grapevine-specific subgroups, as already observed in rice^12^. However, the presence of one large group of grapevine ATL genes suggested the specific evolutionary expansion of this particular subgroup. About 78% of the ATL genes contained between zero and three introns, and the complexity of gene structure correlated with the amino acid sequence similarity in the RING-H2 domain (Supplementary Figure 3). No such relationship was observed at the level of the entire coding region, suggesting that the sequence and function of the RING-H2 domain drove the expansion of this gene family.

The distribution of genes on different chromosomes and the clustering found in certain chromosome segments suggests that the evolution of the grapevine ATL gene family has been driven by duplication events, a frequently-reported phenomenon in plants^55^. Whole-genome duplication and segmental duplication events are the primary origin of new gene functions during the evolution of plants, allowing an increase in biological complexity^56^. Accordingly, several of the grapevine ATL genes appear to originate from tandem duplication events and others from whole-genome duplication, and the retention of the duplicated genes probably reflects the fixation of adaptive mutations that have diversified the corresponding protein functions (subfunctionalisation). A high level of duplication retention in the ATL family is also reported in Arabidopsis, suggesting that the expansion of the ATL family may have been driven by the need to respond to diverse stimuli affecting multiple physiological and pathophysiological processes^13^,^16^.

The hypothesis outlined above was supported by the clustering of expression profiles, which only occasionally mirrored the phylogenetic relationship among the ATL genes (typically only when the genes are tandem duplicates). The expression profiles were informative on a broader level because several ATL genes were differentially expressed in the atlas of grapevine organ development or in defence-related and stress-related gene datasets, with minimal correlation between sequence similarity and expression profiles. For example, even duplicated (paralogous) genes, irrespective of duplication type, showed different expression profiles in different grapevine organs or during plant development, such as the couples VviATL126 (VIT_12s0028g01570) and VviATL128 (VIT_12s0028g01580), VviATL138 (VIT_03s0038g03930) and VviATL139 (VIT_18s0122g00870), or VviATL155 (VIT_07s0005g00710) and VviATL156 (VIT_05s0077g01970).

In the same way, some ATL genes were strongly modulated by biotic stress, e.g. VviATL55 (VIT_07s0191g00230) and VviATL156 (VIT_05s0077g01970), whereas their paralogues VviATL54a (VIT_18s0001g06640) and VviATL155 (VIT_07s0005g00710) respectively, were not, indicating a different role for otherwise similar genes both during development and in response to pathogens. Similarly, in the response to abiotic stress, about half of the paralogous couples showed different or even opposite expression profiles, e.g. the paralogues VviATL120 (VIT_13s0019g01960) and VviATL121 (VIT_06s0004g05080), which were respectively upregulated and downregulated by carbon starvation, in line with the hypothesised functional diversification.

The analysis of expression profiles during grapevine development highlighted some ATL proteins with a putative role in the different physiological conditions we examined. During grapevine development, genes in clusters A and E of the expression atlas were particularly interesting because they featured opposing expression profiles in juvenile and mature tissues (Fig. 5). This supports the global transcriptomic reprogramming associated with the transition from young/green to mature/woody tissues^43^ and suggests that two broad panels of ATL genes may be involved in this process. Interestingly, VviATL23a (VIT_18s0001g01060) in cluster A was previously identified as a “switch” gene controlling the transcriptional shift to maturation by acting as a negative regulator of vegetative metabolic processes such as photosynthesis and cell proliferation^57^. Another interesting example is VviATL105 (VIT_13s0064g01030), which is much more strongly expressed in buds and flowers than other organs, but is downregulated in flowers in response to carbon starvation, suggesting a positive role of this ATL particularly in development, especially reproduction rather than stress responses. Furthermore, a small group of ATL genes in cluster C was consistently upregulated in the rachis, tendrils and leaves from veraison to ripening, whereas most of the ATLs in cluster B were downregulated in leaves undergoing senescence, suggesting a possible association with pathways that are suppressed during senescence. In other plant species, some ATL proteins have been shown to regulate different aspects of plant development such as Arabidopsis ATL25, ATL32 and ATL62 (DNF), and rice EL5^28^,^29^,^30^,^31^. Clear grapevine counterparts of these genes were not identified by our analysis.

The characterisation of ATL gene expression in defence-related datasets indicated that most genes were modulated in a statistically significant manner by pathogens, supporting the hypothesis that ATL genes encode stress-related E3 ubiquitin ligases. More specifically, our survey highlighted a group of 12 ATL genes that were particularly responsive to pathogens, especially the biotrophic pathogens responsible for powdery and downy mildew (compatible and incompatible interactions), and to *B. cinerea* during the development of both grey mould and noble rot. The wider experiment tested the responses of a number of grapevine accessions and species to the powdery mildew pathogen *E. necator*^58^. Interestingly, all resistant accessions contained simple sequence repeat markers related to the Ren-1 resistance gene^59^, but the modulation of selected ATL genes was not always comparable, suggesting that different mechanisms may regulate the response to infection downstream of Ren-1^58^. In contrast, the induction of nine of these 12 ATL genes in response to *P. viticola* occurred solely in the resistant genotype and was detected much earlier^41^, whereas the only available experiment related to herbivore infestation^60^ showed a specific modulation of most ATL transcripts only in response to adapted spider mites and not towards the non-adapted strain.

Arabidopsis counterparts of certain pathogen-responsive grapevine ATL genes have been functionally characterised, including AtATL2, the orthologue of VviATL156 (VIT_05s0077g01970)^17^,^61^, and AtATL55, the orthologue of VviATL55 (VIT_07s0191g00230) (At5g10380), which regulates pathogen responses and programed cell death^62^. Another interesting example is VvATL146 (VIT_14s0128g00120), which is induced by both powdery and downy mildew infections, and is highly similar to Arabidopsis ATL31, ATL6 and ATL9^21^,^63^,^64^,^65^. AtATL31 and its closest homolog AtATL6 regulate pathogen responses and the carbon/nitrogen balance^29^,^64^. Similarly VviATL162 (VIT_07s0005g03120) and VviATL163 (VIT_05s0049g00480), both modulated by pathogens, are the closest homologues of AtATL1, which is repressed by EDR1-mediated phosphorylation to suppress programed cell death^51^. The ATL genes that respond most strongly to pathogens are not restricted to certain chromosomes or phylogenetic clusters and do not show a particular expression profile during organ development.

In the context of abiotic stress, we identified groups of ATL genes that responded more intensely to specific forms of stress (Fig. 7, clusters A, C and E), but the expression profiles were diverse, thus highlighting the existence of specific stress-related responses at the gene level rather than broad responses involving clusters of the ATL family in general. Despite evidence for the involvement of UPS and E3 ubiquitin ligases particularly in ABA-dependent stress responses, few reports attribute such a role to ATL proteins. Examples include Arabidopsis ATL43^12^ and ATL78^26^, as well as soybean GmRFP1^27^. Interestingly, we found that VviATL43, the orthologue of AtATL43, is specifically modulated in response to carbon starvation and not by pathogens or other stimuli. When comparing the list of ATL transcripts modulated by biotic and/or abiotic stresses, we identified a subset of 41 ATLs broadly responsive to both stress categories, whereas two subsets of 21 and 13 ATLs responded more specifically to biotic or abiotic stresses, respectively. Accordingly, the top-12 pathogen-responsive genes also responded to one or more abiotic challenges, except VviATL55 and VviATL123, which responded specifically to biotic stress.

## Conclusion

The grapevine ATL family comprises 96 members with diverse sequences that may be necessary for ligase specificity and the regulation of different metabolic processes. Although we found that some members of the family appear to be developmentally regulated and others preferentially respond to pathogens or abiotic stress, there was no clear distinction between the two broad functions. This may reflect the overlap among different signalling pathways, particularly those regulated by hormones, which can be involved both in development and defence^66^,^67^. Few ATL genes have been functionally characterised in Arabidopsis or other species, and most functional data are derived from the analysis of gene expression in response to elicitors such as flagellin and chitin^11^,^17^. The functions of ATL genes in Arabidopsis and grapevine appear to be at least partly conserved, providing useful candidate genes for further functional characterisation in the context of biotic and abiotic stress responses.

## Methods

### Identification and annotation of putative grapevine ATL genes

The translated *Vitis vinifera* cv. Pinot Noir genome sequence (PN40024 12X assembly V1 prediction) was obtained from the Grapevine Genome CRIBI Biotech Centre website (http://genomes.cribi.unipd.it/grape/)^45^, accounting for a total of 29971 predicted sequences. The amino acid sequences of the RING-H2 domain from the eight predicted ATLs identified previously^41^, that contain the canonical CxxC(13x)PxCxHxxHxxCxxxW(7x)CxxCW motif^12^, were used to define a position-specific scoring matrix (PSSM). This profile was used as a PSI-BLAST query (http://blast.ncbi.nlm.nih.gov/blast.cgi)^68^ against all predicted grapevine proteins. Two PSI-BLAST iterations were performed using a BLOSUM62 matrix and an e-value threshold of 0.001. The searches converged after the second iteration, 143 proteins were retrieved and the corresponding sequences were aligned using MUSCLE (http://www.ebi.ac.uk/Tools/msa/muscle/)^69^ with default parameters in MEGA v5 (http://www.megasoftware.net/)^70^. The sequences were visually inspected to exclude incorrectly predicted ATLs. Only 96 proteins containing a canonical RING-H2 domain with correctly spaced Zn-coordinating cysteine and histidine residues and a proline residue before the third cysteine were retained^12^. Multiple Em for Motif Elicitation (MEME) (http://meme.nbcr.net/meme/)^71^ was used to define the specific LOGO motif of the RING-H2 domain whereas the GDL motif LOGO was generated with Geneious v9.1.4 (http://www.geneious.com)^72^.

Physical parameters, including the isoelectric point (pI) and molecular weight (kDa), were calculated using ProtParam on the ExPasy website (http://web.expasy.org/protparam/)^73^. Protein subcellular location was predicted using ngLOC v1.0 (http://genome.unmc.edu/ngLOC/index.html)^74^ with default settings, TargetP v1.1 (http://www.cbs.dtu.dk/services/TargetP/)^75^ and Protein Prowler Subcellular Localisation v1.2 (http://bioinf.scmb.uq.edu.au:8080/pprowler_webapp_1-2/)^76^ with a cut-off of probability of 0.5. Phosphorylation sites were predicted using MUsite v1.0 (http://musite.sourceforge.net/)^77^.

### Analysis of additional protein domains

The Pfam database was used to identify additional conserved motifs outside the RING-H2 domain, with a cut-off value of 1.0 to identify significant matches. The presence of putative transmembrane regions was investigated using TMHMM Server v2.0 from the Center for Biological Sequence Analysis (http://www.cbs.dtu.dk/services/TMHMM/)^78^. Proteins without predicted transmembrane domains were analysed with ProtScale on the Expasy website (http://web.expasy.org/protscale/)^73^ using the method of Kyte and Doolittle^79^ in order to identify hydrophobic regions.

### Chromosomal distribution, duplications and exon–intron organisation

All grapevine ATL genes were mapped to chromosomes based on the information present on the Grapevine Genome CRIBI Biotech Centre website. ATL collinear paralogues were identified using MCScanX^80^. Briefly, all predicted grapevine proteins were self-compared using BLASTP^81^ and for a protein sequence the best five non-self matches with an E value threshold of 1 × 10^−5^ were reported. These hits were studied according to the position of the genes on the chromosomes and scaffolds (V1 gene prediction). The highest scoring path was identified by dynamic programming with standard settings^80^. ATL loci were classified as dispersed, tandem/proximal or segmental/whole-genome duplications based on the number of matching hits and positions in chromosomes and scaffolds. Enrichment analysis was performed using Fisher’s exact test with a null hypothesis of no association between the members of the ATL family and a specified gene duplication mode. The resulting p-values were corrected using the Bonferroni method to accommodate bias due to multiple comparisons.

The chromosomal distribution and duplication state of the genes was visualised using PhenoGram (http://visualization.ritchielab.psu.edu/phenograms/plot - © 2012 Ritchie Lab)^82^. The predicted exon–intron structure was retrieved from the V1 annotation of the grapevine genome (http://genomes.cribi.unipd.it/grape/) and represented using the Interactive Tree of Life website version 3.2.4 (http://itol.embl.de/)^83^.

### Phylogenetic analysis and nomenclature of the ATL gene family

All phylogenetic trees were constructed using Phylogeny.fr (http://www.phylogeny.fr/) and represented using the Interactive Tree of Life website^83^. The nomenclature of the 96 grapevine ATL genes was determined by comparison with the 83 Arabidopsis ATL nucleotide sequences retrieved from UniProt (http://www.uniprot.org), following the rules established by the Grapevine Super Nomenclature Committee^44^. Due to the high variability among gene sequences, relaxed settings were applied to the GBlocks (v0.91b)^84^ curation following multiple sequence alignment using MUSCLE (http://www.ebi.ac.uk/Tools/msa/muscle/)^69^. An unrooted maximum-likelihood phylogenetic tree was then constructed using PhyML v3.0 (http://www.atgc-montpellier.fr/phyml/)^85^. Bootstrap values below 70% were collapsed.

### Spatiotemporal expression profiling during grapevine development

The expression profiles of the grapevine ATL genes were analysed in the *Vitis vinifera* cv. Corvina gene expression atlas of different organs at various developmental stages^43^. Microarray data were obtained from the Gene Expression Omnibus website entry GSE36128 (http://www.ncbi.nlm.nih.gov/geo/query/acc.cgi ? acc=GSE36128/geo/). Expression data were analysed and graphically represented with MultiExperiment Viewer v4.9 (http://www.tm4.org/mev.html) after normalisation based on the median expression value of each gene in all tissues and organs. The co-expression of grapevine ATL genes was calculated using the weighted Pearson co-expression coefficient (PCC) as previously described^86^. The co-expression data matrix was compared to the pairwise alignment score matrix using a Mantel correlation test among distance matrices^87^.

### Expression profiling in response to biotic and abiotic stresses

Robust multi-array (RMA) normalised microarray data and raw counts from next-generation sequencing data (RNA-seq) were obtained from the Gene Expression Omnibus (GEO) and ArrayExpress databases (https://www.ebi.ac.uk/arrayexpress/). Experiments included grapevine plants infected with *Grapevine rupestris stem pitting-associated virus* (GRSPaV)^88^, *Botrytis cinerea*^89^,^90^, *Erysiphe necator*^58^, *Neofusicoccum parvum*^91^, *Plasmopara viticola*^41^,^92^, *Eutypa lata* (Unpublished Microarray Nimblegen), or infested with the spider mite *Tetranyhus urticae*^60^, and grapevine plants subjected to different abiotic stresses, namely drought stress in cv. Tocai berries during development and ripening^93^, berry cultures treated with exogenous glucose^94^, heat stress/acclimation in berries during berry development and ripening^95^, carbon starvation due to plant shading at bloom in flowers/inflorescences^96^ and UV-C treatment of berry skins^97^. Differential gene expression re-analysis based on the microarray and RNA-seq data was carried out using *limma*^98^ and *DESeq2*^99^, respectively, at FDR values < 0.05. Log_2_ FC >| 0.5| was used as the threshold for differential expression between any two conditions (e.g. infected versus control).

## Additional Information

**How to cite this article**: Ariani, P. *et al*. Genome-wide characterisation and expression profile of the grapevine ATL ubiquitin ligase family reveal biotic and abiotic stress-responsive and development-related members. *Sci. Rep.* **6**, 38260; doi: 10.1038/srep38260 (2016).

**Publisher's note:** Springer Nature remains neutral with regard to jurisdictional claims in published maps and institutional affiliations.

## Supplementary Material

Supplementary Figures and Tables

Supplementary Table 2

Supplementary Table 3

## Figures and Tables

**Table 1:** ATL genes in the *V. vinifera* genome and sequence characteristics of the corresponding proteins.

Name	Gene ID	Gene length (bp)	Intron number	UniProt ID	Protein length (aa)	RING-H2 motif	TM/H domain number	Other domains
VviATL3	VIT_09s0002g00220	1245	0	F6HXK6	304	PxC	1	
VviATL4[VviRHX1A]	VIT_15s0021g00890	1827	3	D7SM36	203	PxC	0	
VviATL18	VIT_11s0118g00780	1113	2	F6HCI8	193	PC	0	
VviATL23a	VIT_18s0001g01060	935	0	F6H0E4	114	PxC	0.5	
VviATL23b	VIT_18s0001g01050	399	0	E0CQX3	132	PxC	1	
VviATL24	VIT_17s0000g06460	4466	4	D7SI89	217	PxC	1	
VviATL27	VIT_00s0264g00020	2554	4	D7T1R5	235	PxC	1	
VviATL43	VIT_11s0052g00530	1576	2	D7SQD9	457	PxC	3	
VviATL54a	VIT_18s0001g06640	3221	1	F6H0Y5	405	PxC	1	
VviATL54b	VIT_03s0017g00670	2774	1	F6HTI0	427	PxC	1	
VviATL55[VviRING1]	VIT_07s0191g00230	1844	0	F6HRP9	372	PxC	1	
VviATL63	VIT_06s0004g06930	804	0	D7SJU6	267	PxC	1	
VviATL65	VIT_03s0063g01890	2068	0	F6HQI8	396	PxC	1	
VviATL82	VIT_01s0026g02540	820	0	F6HPQ9	233	PC	0.5	
VviATL83	VIT_17s0000g08400	1887	0	F6GSQ4	143	PC	0	
VviATL84	VIT_06s0004g00120	1853	0	F6GUP5	368	PC	0.5	zf-RING_3
VviATL85	VIT_12s0034g01400	786	0	F6H965	261	PC	0.5	
VviATL86	VIT_12s0034g01390	1434	1	D7T016	451	PC	0.5	
VviATL87	VIT_18s0001g03270	1002	0	F6H0T2	333	PC	0.5	zf-RING_3
VviATL88	VIT_08s0040g00590	1320	0	F6HQR2	314	PC	0	zf-RING_3
VviATL89	VIT_06s0009g02350	4862	0	F6HAD8	336	PC	0	zf-RING_3
VviATL90	VIT_06s0004g05090	1728	0	F6GUZ2	386	PC	0	zf-RING_3; DUF1117
VviATL91	VIT_13s0019g01980	11750	1	F6HNV7	763	PC	0	zf-RING_3; DUF1117; Asp
VviATL92	VIT_08s0007g00720	6094	2	F6HL86	516	PC	0.5	zf-RING_3; DUF1117
VviATL93	VIT_13s0156g00140	3799	0	F6HPS6	312	PC	0	zf-RING_3
VviATL94	VIT_17s0053g00320	1165	0	F6HVS8	369	PC	0	zf-RING_3
VviATL95	VIT_04s0008g02290	2454	4	D7SU02	293	PC	0.5	
VviATL96	VIT_08s0056g00320	7370	2	F6HMS0	590	PC	1	
VviATL97	VIT_11s0016g03190	925	0	D7TBH2	168	PC	0.5	
VviATL98	VIT_17s0000g04730	1691	0	F6GTF6	439	PC	0	
VviATL99	VIT_14s0083g00710	1285	0	F6GVT7	391	PC	0	
VviATL100	VIT_08s0040g02950	972	0	F6HQW4	285	PC	0.5	
VviATL101	VIT_18s0001g14530	2804	0	A5BX64	334	PC	0	zf-RING_3
VviATL102	VIT_02s0012g01440	1720	0	F6HT83	292	PxC	0	
VviATL103	VIT_19s0090g00400	1594	4	F6HEK0	220	PxC	1	
VviATL104	VIT_05s0051g00730	19438	5	F6HS63	190	PC	0	
VviATL105	VIT_13s0064g01030	4567	6	D7T2Z6	247	PC	0	
VviATL106	VIT_06s0004g01930	9252	6	D7SL69	252	PC	0	
VviATL107	VIT_18s0075g00220	25207	4	F6GY85	444	PC	1	PA
VviATL108	VIT_04s0023g03580	4487	8	F6GWM0	422	PC	1	PA; Rhodanese
VviATL109	VIT_11s0052g00360	396	0	D7SQF4	131	PxC	0	
VviATL110	VIT_11s0118g00760	8334	5	F6HCI6	542	PC	0	
VviATL111	VIT_02s0087g00420	10368	5	F6HJ39	561	PC	0	
VviATL112	VIT_19s0015g01000	3045	2	D7UAM0	343	PC	4	
VviATL113	VIT_11s0016g04450	4562	3	D7TBT7	407	PC	4	
VviATL114	VIT_04s0008g04280	8051	3	D7SUI3	401	PC	4	
VviATL115	VIT_09s0002g05120	6212	3	F6HX40	442	PC	4	
VviATL116	VIT_09s0002g05140	1686	3	F6HX42	309	PC	4	
VviATL117	VIT_09s0002g05130	950	2	F6HX41	208	PC	2	
VviATL118	VIT_12s0057g01330	971	2	F6HHR0	202	PC	0.5	
VviATL119	VIT_08s0040g00310	24289	3	F6HQS8	385	PC	4	
VviATL120	VIT_13s0019g01960	8052	7	D7TLR3	275	PC	2	
VviATL121	VIT_06s0004g05080	4108	7	F6GUZ3	284	PC	0.5	
VviATL122	VIT_17s0000g08210	7519	10	F6GSR5	433	PC	4	
VviATL123	VIT_03s0091g00480	3492	2	D7SXT7	278	PC	0	
VviATL124	VIT_04s0023g03460	576	0	A5B1V6	166	PxC	1	
VviATL125	VIT_00s0349g00040	562	0	F6H5I9	135	PC	0.5	
VviATL126	VIT_12s0028g01580	1624	2	F6H540	339	PC	0	
VviATL127	VIT_12s0028g01560	1080	2	F6H541	190	PC	0	
VviATL128	VIT_12s0028g01570	1440	2	E0CTW4	224	PC	0.5	
VviATL129	VIT_15s0048g02030	4833	4	F6I314	382	PC	5	
VviATL130	VIT_02s0025g04150	7750	4	F6HUF7	384	PC	5	
VviATL131	VIT_15s0048g01840	2566	2	D7U7K1	201	PxC	1	
VviATL132	VIT_13s0074g00370	8204	2	D7UBU6	209	PxC	0.5	
VviATL133	VIT_18s0089g00860	3502	2	D7SMW5	221	PxC	1	
VviATL134	VIT_06s0004g08080	1766	1	F6GUB5	263	PxC	1	
VviATL135	VIT_08s0058g01270	1007	0	F6GXX0	195	PxC	1	
VviATL136	VIT_15s0024g01990	414	0	F6I5A3	137	PxC	0	
VviATL137	VIT_13s0067g02880	1008	0	A5BMU1	197	PxC	1	
VviATL138	VIT_03s0038g03930	542	0	F6I0R1	129	PxC	1	
VviATL139	VIT_18s0122g00870	794	0	F6I6U9	184	PxC	1	
VviATL140	VIT_07s0031g02250	872	0	D7SWC6	182	PxC	1	
VviATL141	VIT_11s0065g01210	696	0	F6H9R6	167	PxC	1	
VviATL142	VIT_15s0046g02070	1042	0	F6I654	197	PxC	1	
VviATL143	VIT_13s0084g00140	753	1	D7TX41	191	PxC	1	
VviATL144	VIT_02s0025g01430	3689	1	D7TVF6	386	PxC	1	GUB_WAK_bind
VviATL145	VIT_16s0098g00250	2508	1	F6H7E1	367	PxC	1	
VviATL146	VIT_15s0046g00930	1884	1	D7UCP8	372	PxC	1	WAK_assoc
VviATL147	VIT_18s0001g06670	1321	0	F6H0Y6	398	PxC	2	
VviATL148	VIT_14s0128g00120	1618	0	A5BY68	420	PxC	2	
VviATL149	VIT_12s0028g02530	1016	0	F6H4 × 9	254	PxC	1	
VviATL150	VIT_10s0003g00850	1020	0	F6HM71	218	PxC	1	
VviATL151	VIT_11s0016g03420	9067	1	F6HH04	469	PxC	2	
VviATL152	VIT_11s0037g01400	1869	0	F6HYP7	543	PxC	1	
VviATL153	VIT_01s0026g00300	1412	2	F6HPM6	420	PxC	1	
VviATL154	VIT_14s0066g01610	1739	0	F6HV15	386	PxC	1	
VviATL155	VIT_07s0005g00710	1013	0	F6HZ62	263	PxC	1	
VviATL156	VIT_05s0077g01970	1374	0	F6H6W1	317	PxC	1	
VviATL157	VIT_13s0019g01020	480	0	F6HN68	159	PxC	1	
VviATL158	VIT_13s0019g01000	453	0	F6HN69	150	PxC	1	
VviATL159	VIT_13s0019g00990	503	0	F6HN70	167	PxC	1	
VviATL160	VIT_09s0002g01500	806	0	F6HXY3	140	PxC	1	
VviATL161	VIT_11s0016g01430	651	0	F6HGU1	178	PxC	1	
VviATL162	VIT_07s0005g03120	917	1	F6HZI2	264	PxC	1	
VviATL163	VIT_05s0049g00480	1694	1	F6H8L8	390	PxC	2	
VviATL164	VIT_15s0045g00330	1034	0	D7U5P0	338	PxC	1	

TM: transmembrane; H: hydrophobic; 0.5 indicates the presence of one or more hydrophobic regions.
