# Supplementary material for: Genome-wide characterisation and expression profile of the grapevine ATL ubiquitin ligase family reveal biotic and abiotic stress-responsive and development-related members
Source: Sci Rep. 2016 Dec 2;6:38260. doi: 10.1038/srep38260 (PMC5133618; doi:10.1038/srep38260)
Supplement: Supplementary Figures and Tables [file srep38260-s1.pdf]

Genome-wide characterisation and expression profile of the grapevine ATL ubiquitin ligase family reveal biotic and abiotic stress-responsive and development-related members

Pietro Ariani[1], Alice Regaiolo[1], Arianna Lovato[1], Alejandro Giorgetti[1], Andrea Porceddu[2], Salvatore Camiolo[2], Darren Wong[3], Simone Castellarin[3], Elodie Vandelle[1*] and Annalisa Polverari[1*]

[1] Dipartimento di Biotecnologie, Università degli Studi di Verona, Strada Le Grazie 15, Verona, 37134, Italy

[2] Università degli Studi di Sassari, Dipartimento di Agraria, SACEG, Via Enrico De Nicola 1, Sassari, 07100, Italy

[3] Wine Research Centre, University of British Columbia, 326 – 2205 East Mall, Vancouver, BC V6T 1Z4, Canada

*corresponding authors: Annalisa Polverari (annalisa.polverari@univr.it), Elodie Vandelle (elodiegenevieve.vandelle@univr.i.t).

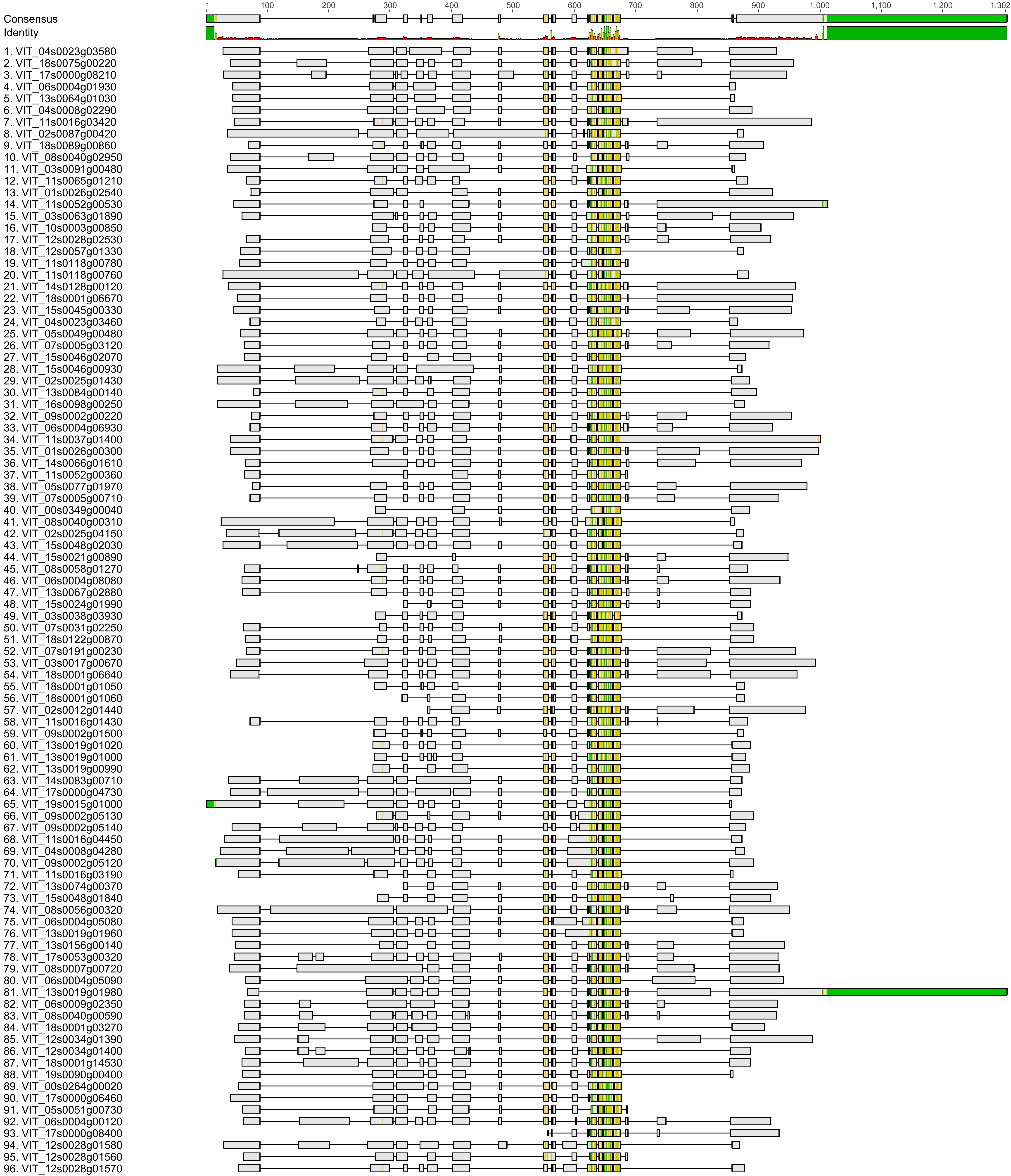
Consensus
Identity
1
100
200
300
400
500
600
700
800
900
1,000
1,100
1,200
1,302
1. VIT_04s0023g03580
2. VIT_18s0075g00220
3. VIT_17s0000g08210
4. VIT_06s0004g01930
5. VIT_13s0064g01030
6. VIT_04s0008g02290
7. VIT_11s0016g03420
8. VIT_02s0087g00420
9. VIT_18s0089g00860
10. VIT_08s0040g02950
11. VIT_03s0091g00480
12. VIT_11s0065g01210
13. VIT_01s0026g02540
14. VIT_11s0052g00530
15. VIT_03s0063g01890
16. VIT_10s0003g00850
17. VIT_12s0028g02530
18. VIT_12s0057g01330
19. VIT_11s0118g00780
20. VIT_11s0118g00760
21. VIT_14s0128g00120
22. VIT_18s0001g06670
23. VIT_15s0045g00330
24. VIT_04s0023g03460
25. VIT_05s0049g00480
26. VIT_07s0005g03120
27. VIT_15s0046g02070
28. VIT_15s0046g00930
29. VIT_02s0025g01430
30. VIT_13s0084g00140
31. VIT_16s0098g00250
32. VIT_09s0002g00220
33. VIT_06s0004g06930
34. VIT_11s0037g01400
35. VIT_01s0026g00300
36. VIT_14s0066g01610
37. VIT_11s0052g00360
38. VIT_05s0077g01970
39. VIT_07s0005g00710
40. VIT_00s0349g00040
41. VIT_08s0040g00310
42. VIT_02s0025g04150
43. VIT_15s0048g02030
44. VIT_15s0021g00890
45. VIT_08s0058g01270
46. VIT_06s0004g08080
47. VIT_13s0067g02880
48. VIT_15s0024g01990
49. VIT_03s0038g03930
50. VIT_07s0031g02250
51. VIT_18s0122g00870
52. VIT_07s0191g00230
53. VIT_03s0017g00670
54. VIT_18s0001g06640
55. VIT_18s0001g01050
56. VIT_18s0001g01060
57. VIT_02s0012g01440
58. VIT_11s0016g01430
59. VIT_09s0002g01500
60. VIT_13s0019g01020
61. VIT_13s0019g01000
62. VIT_13s0019g00990
63. VIT_14s0083g00710
64. VIT_17s0000g04730
65. VIT_19s0015g01000
66. VIT_09s0002g05130
67. VIT_09s0002g05140
68. VIT_11s0016g04450
69. VIT_04s0008g04280
70. VIT_09s0002g05120
71. VIT_11s0016g03190
72. VIT_13s0074g00370
73. VIT_15s0048g01840
74. VIT_08s0056g00320
75. VIT_06s0004g05080
76. VIT_13s0019g01960
77. VIT_13s0156g00140
78. VIT_17s0053g00320
79. VIT_08s0007g00720
80. VIT_06s0004g05090
81. VIT_13s0019g01980
82. VIT_06s0009g02350
83. VIT_08s0040g00590
84. VIT_18s0001g03270
85. VIT_12s0034g01390
86. VIT_12s0034g01400
87. VIT_18s0001g14530
88. VIT_19s0090g00400
89. VIT_00s0264g00020
90. VIT_17s0000g06460
91. VIT_05s0051g00730
92. VIT_06s0004g00120
93. VIT_17s0000g08400
94. VIT_12s0028g01580
95. VIT_12s0028g01560
96. VIT_12s0028g01570

Supplementary Figure 1. Protein structure of the 96 grapevine ATLs. The alignment was generated with Geneious (MUSCLE algorithm) using the protein sequences of the 96 ATLs identified in whole Vitis vinifera genome.

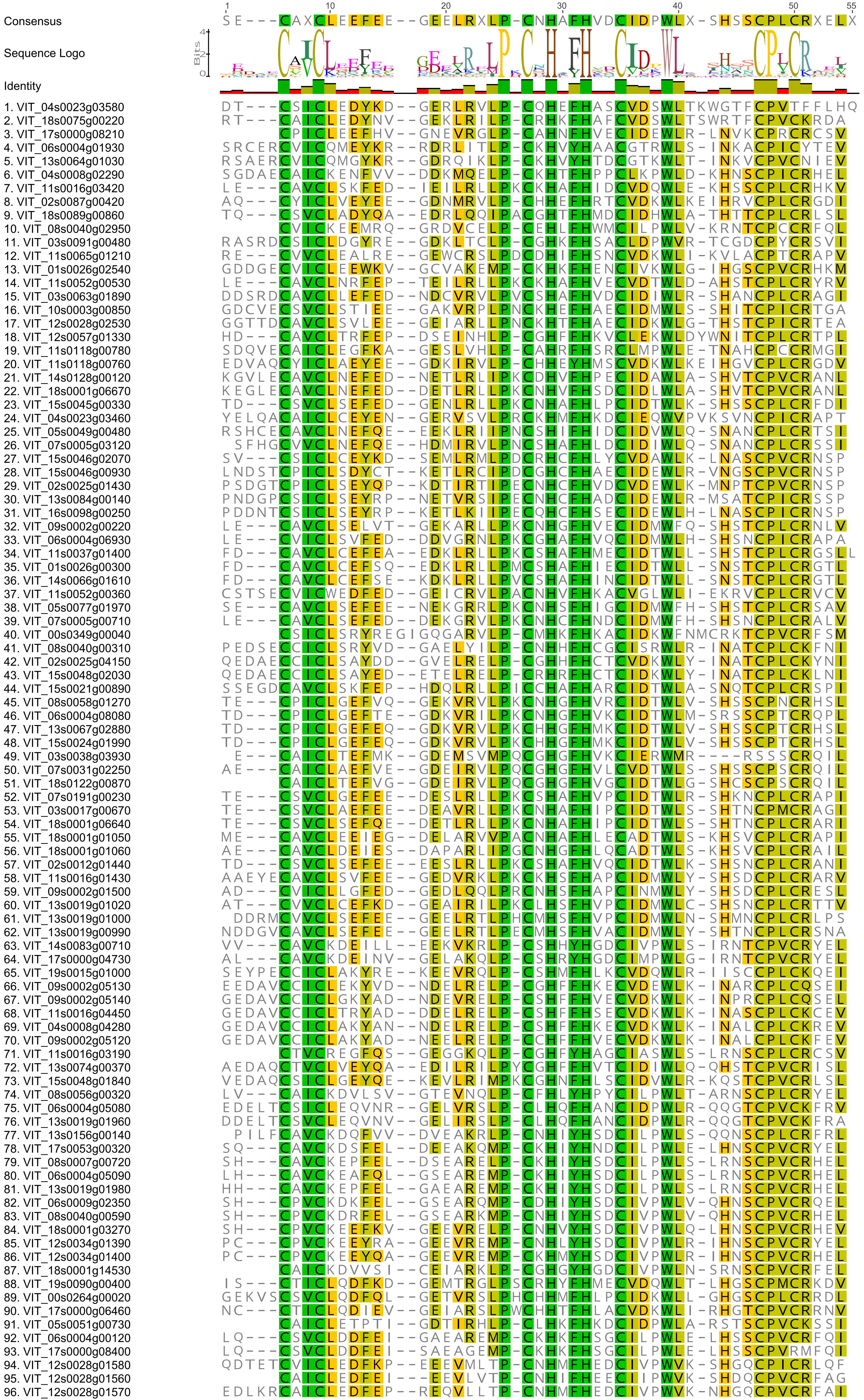

Consensus
Sequence Logo
Identity
Bits
1. VIT_04s0023g03580
2. VIT_18s0075g00220
3. VIT_17s0000g08210
4. VIT_06s0004g01930
5. VIT_13s0064g01030
6. VIT_04s0008g02290
7. VIT_11s0016g03420
8. VIT_02s0087g00420
9. VIT_18s0089g00860
10. VIT_08s0040g02950
11. VIT_03s0091g00480
12. VIT_11s0065g01210
13. VIT_01s0026g02540
14. VIT_11s0052g00530
15. VIT_03s0063g01890
16. VIT_10s0003g00850
17. VIT_12s0028g02530
18. VIT_12s0057g01330
19. VIT_11s0118g00780
20. VIT_11s0118g00760
21. VIT_14s0128g00120
22. VIT_18s0001g06670
23. VIT_15s0045g00330
24. VIT_04s0023g03460
25. VIT_05s0049g00480
26. VIT_07s0005g03120
27. VIT_15s0046g02070
28. VIT_15s0046g00930
29. VIT_02s0025g01430
30. VIT_13s0084g00140
31. VIT_16s0098g00250
32. VIT_09s0002g00220
33. VIT_06s0004g06930
34. VIT_11s0037g01400
35. VIT_01s0026g00300
36. VIT_14s0066g01610
37. VIT_11s0052g00360
38. VIT_05s0077g01970
39. VIT_07s0005g00710
40. VIT_00s0349g00040
41. VIT_08s0040g00310
42. VIT_02s0025g04150
43. VIT_15s0048g02030
44. VIT_15s0021g00890
45. VIT_08s0058g01270
46. VIT_06s0004g08080
47. VIT_13s0067g02880
48. VIT_15s0024g01990
49. VIT_03s0038g03930
50. VIT_07s0031g02250
51. VIT_18s0122g00870
52. VIT_07s0191g00230
53. VIT_03s0017g00670
54. VIT_18s0001g06640
55. VIT_18s0001g01050
56. VIT_18s0001g01060
57. VIT_02s0012g01440
58. VIT_11s0016g01430
59. VIT_09s0002g01500
60. VIT_13s0019g01020
61. VIT_13s0019g01000
62. VIT_13s0019g00990
63. VIT_14s0083g00710
64. VIT_17s0000g04730
65. VIT_19s0015g01000
66. VIT_09s0002g05130
67. VIT_09s0002g05140
68. VIT_11s0016g04450
69. VIT_04s0008g04280
70. VIT_09s0002g05120
71. VIT_11s0016g03190
72. VIT_13s0074g00370
73. VIT_15s0048g01840
74. VIT_08s0056g00320
75. VIT_06s0004g05080
76. VIT_13s0019g01960
77. VIT_13s0156g00140
78. VIT_17s0053g00320
79. VIT_08s0007g00720
80. VIT_06s0004g05090
81. VIT_13s0019g01980
82. VIT_06s0009g02350
83. VIT_08s0040g00590
84. VIT_18s0001g03270
85. VIT_12s0034g01390
86. VIT_12s0034g01400
87. VIT_18s0001g14530
88. VIT_19s0090g00400
89. VIT_00s0264g00020
90. VIT_17s0000g06460
91. VIT_05s0051g00730
92. VIT_06s0004g00120
93. VIT_17s0000g08400
94. VIT_12s0028g01580
95. VIT_12s0028g01560
96. VIT_12s0028g01570

Supplementary Figure 2. Alignment of grapevine ATL RING-H2 domains. The alignment was generated with Geneious (MUSCLE algorithm) using the protein sequences of the 96 ATLs identified in the whole *Vitis vinifera* genome. The color index indicates the level of amino acid conservation among the 96 sequences, with green color indicating 100% of residue conservation, dark yellow 80 to 100% of conservation, light yellow 60 to 80% of conservation and white a conservation level <60%.

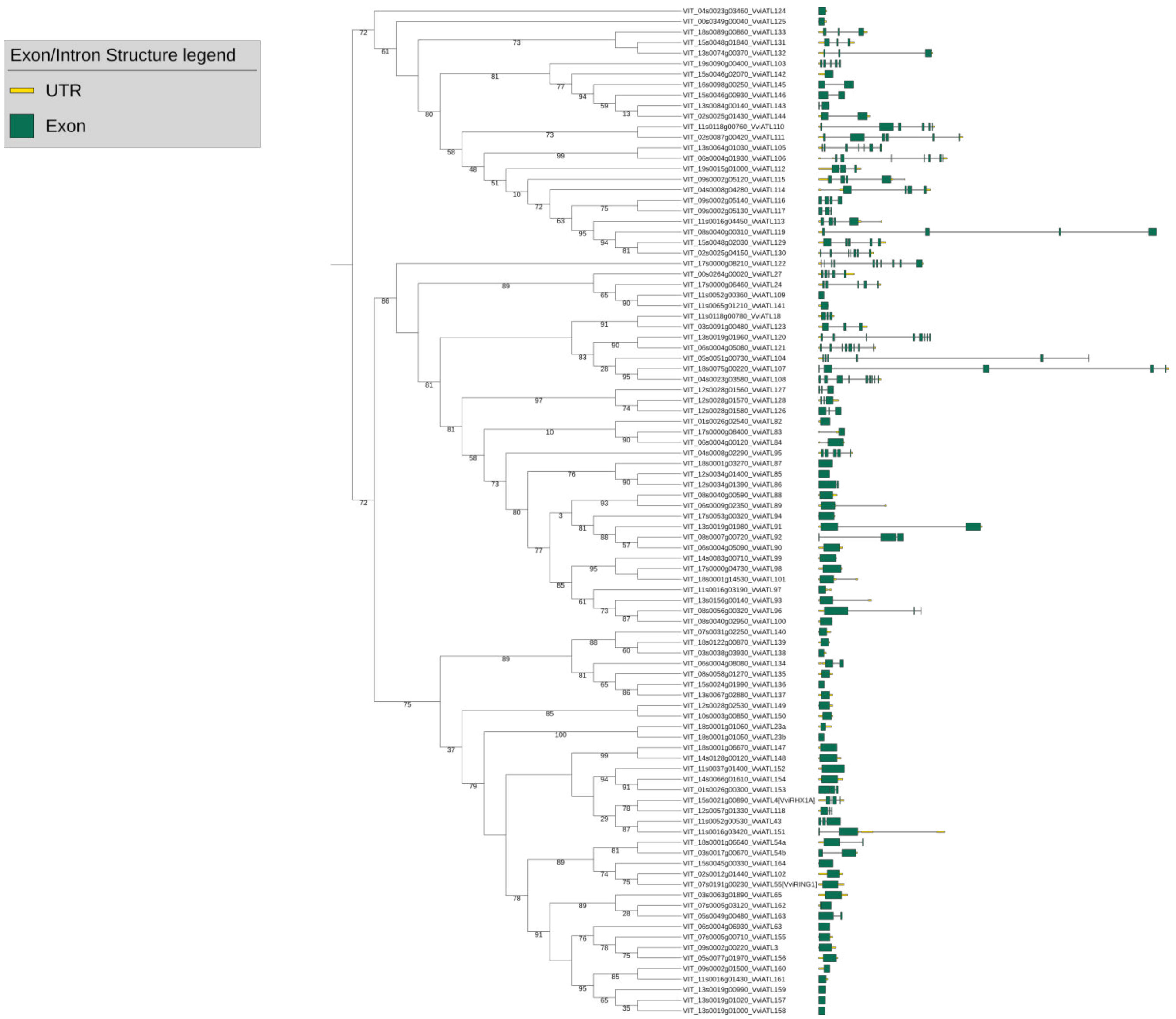

Supplementary Figure 3. Phylogenetic analysis of the grapevine ATL RING-H2 domains and exon-intron organization of the corresponding *ATL* genes. The unrooted tree was generated with the Phylogeny.fr Suite (http://www.phylogeny.fr) using the full-length protein sequences of the 96 grapevine ATLs identified in the whole *Vitis vinifera* genome. Exons (green boxes), introns (grey lines) and untranslated regions (UTRs, yellow boxes) are shown.

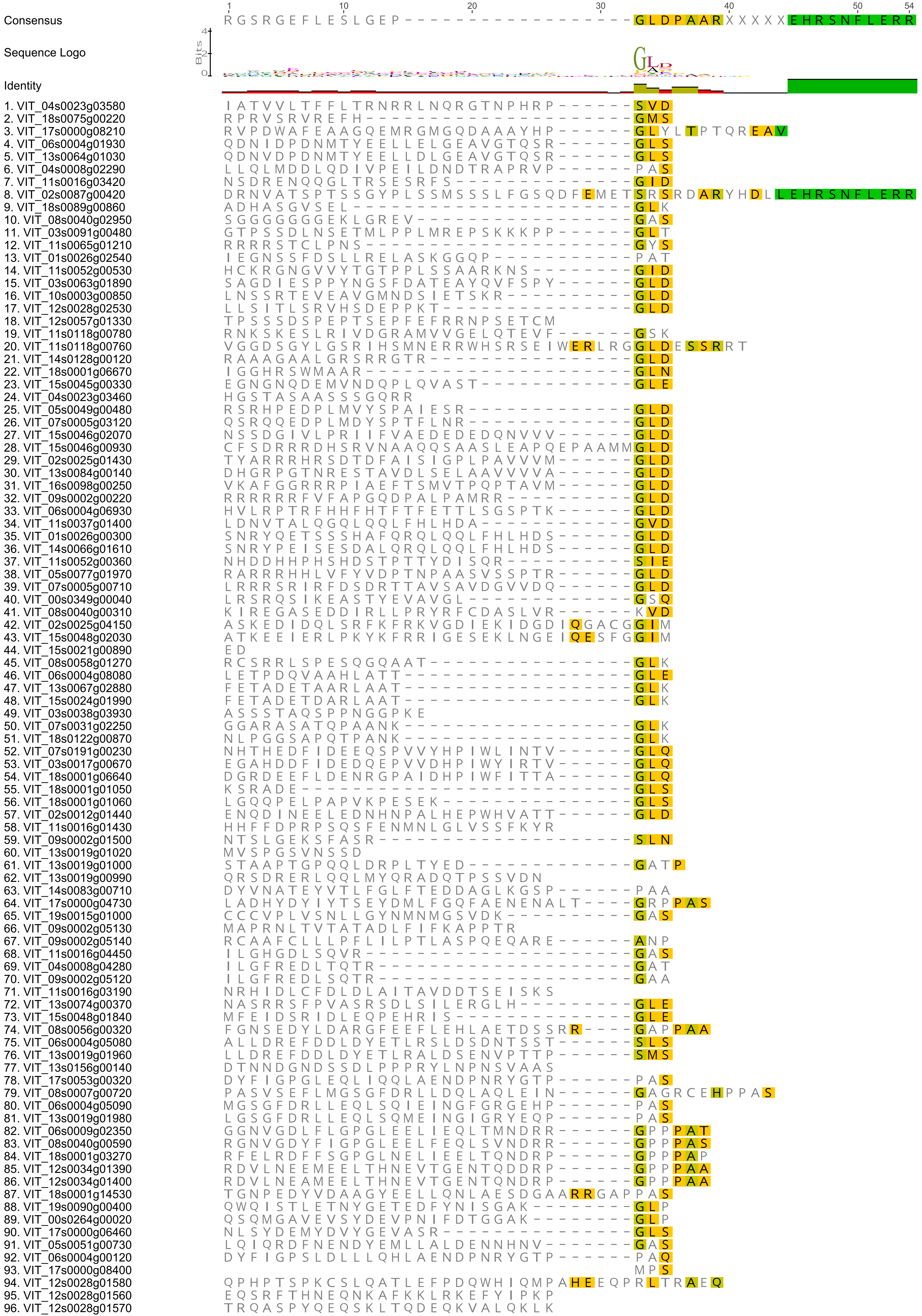
1 10 20 30 40 50 54
Consensus RGSRGEFLESLGEP------------------GLDPAARXXXXXEHRSNFLERR
Sequence Logo
Bits 4 2 0
Identity
1. VIT_04s0023g03580 IATVVLTFFLTRNRRLNQRGTNPHRP------SVD
2. VIT_18s0075g00220 RPRVSRVREFH GMS
3. VIT_17s0000g08210 RVPDWAFEAAGQEMRGMGQDAAAYHP------GLYLTPTQREAV
4. VIT_06s0004g01930 QDNIDPDNMTYEELLELGEAVGTQSR------GLS
5. VIT_13s0064g01030 QDNVDPDNMTYEELLDLGEAVGTQSR------GLS
6. VIT_04s0008g02290 LLQLMDDLQDIVPEILDNDTRAPRVP------PAS
7. VIT_11s0016g03420 NSDRENQQGLTRSESRFS--------------GID
8. VIT_02s0087g00420 DRNVATSPTSSGYPLSSMSSSLFGSQDFEMETSRSRDARYHDLLEHRSNFLERR
9. VIT_18s0089g00860 ADHASGVSEL----------------------GLK
10. VIT_08s0040g02950 SGGGGGGGEKLGREV-----------------GAS
11. VIT_03s0091g00480 GTPSSDLNSETMLPPLMREPSKKKPP------GLT
12. VIT_11s0065g01210 RRRRSTCLPNS---------------------GYS
13. VIT_01s0026g02540 IEGNSSFDSLLRELASKGGQP-----------PAT
14. VIT_11s0052g00530 HCKRGNGVVYTGTPPLSSAARKNS--------GID
15. VIT_03s0063g01890 SAGDIESPPYNGSFDATEAYQVFSPY------GLD
16. VIT_10s0003g00850 LNSSRTEVEAVGMNDSIETSKR----------GLD
17. VIT_12s0028g02530 LLSITLSRVHSDEPPKT---------------GLD
18. VIT_12s0057g01330 TPSSSDSPEPTSEPFEFRRNPSETCM
19. VIT_11s0118g00780 RNKSKESLRIVDGRAMVVGELQTEVF------GSK
20. VIT_11s0118g00760 VGGDSGYLGSRIHSMNERRWHSRSEIWERLRGGLDESSRRT
21. VIT_14s0128g00120 RAAAGAALGRSRRGTR----------------GLD
22. VIT_18s0001g06670 IGGHRSWMAAR---------------------GLN
23. VIT_15s0045g00330 EGNGNQDEMVNDQPLQVAST------------GLE
24. VIT_04s0023g03460 HGSTASAASSSGQRR
25. VIT_05s0049g00480 RSRHPEDPLMVYSPAIESR-------------GLD
26. VIT_07s0005g03120 QSRQQEDPLMDYSPTFLNR-------------GLD
27. VIT_15s0046g02070 NSSDGIVLPRIIFVAEDEDEDQNVVV------GLD
28. VIT_15s0046g00930 CFSDRRRDHSRVNAAQQSAASLEAPQEPAAMMGLD
29. VIT_02s0025g01430 TYARRRHRSDTDFAISIGPLPAVVVM------GLD
30. VIT_13s0084g00140 DHGRPGTNRESTAVDLSELAAVVVVA------GLD
31. VIT_16s0098g00250 VKAFGGRRRPIAEFTSMVTPQPTAVM------GLD
32. VIT_09s0002g00220 RRRRRRFVFAPGQDPALPAMRR----------GLD
33. VIT_06s0004g06930 HVLRPTRFHHFHTFTFETTLSGSPTK------GLD
34. VIT_11s0037g01400 LDNVTALQGQLQQLFHLHDA------------GVD
35. VIT_01s0026g00300 SNRYQETSSSHAFQRQLQQLFHLHDS------GLD
36. VIT_14s0066g01610 SNRYPEISESDALQRQLQQLFHLHDS------GLD
37. VIT_11s0052g00360 NHDDHHPHSHDSTPTTYDISQR----------SIE
38. VIT_05s0077g01970 RARRRHHLVFYVDPTNPAASVSSPTR------GLD
39. VIT_07s0005g00710 LRRRSRIRFDSDRTTAVSAVDGVVDQ------GLD
40. VIT_00s0349g00040 LRSRQSIKEASTYEVAVGL-------------GSQ
41. VIT_08s0040g00310 KIREGASEDDIRLLPRYRFCDASLVR------KVD
42. VIT_02s0025g04150 ASKEDIDQLSRFKFRKVGDIEKIDGDIQGACGGIM
43. VIT_15s0048g02030 ATKEEIERLPKYKFRRIGESEKLNGEIQESFGGIM
44. VIT_15s0021g00890 ED
45. VIT_08s0058g01270 RCSRRLSPESQGQAAT----------------GLK
46. VIT_06s0004g08080 LETPDQVAAHLATT------------------GLE
47. VIT_13s0067g02880 FETADETAARLAAT------------------GLK
48. VIT_15s0024g01990 FETADETDARLAAT------------------GLK
49. VIT_03s0038g03930 ASSSTAQSPPNGGPKE
50. VIT_07s0031g02250 GGARASATQPAANK------------------GLK
51. VIT_18s0122g00870 NLPGGSAPQTPANK------------------GLK
52. VIT_07s0191g00230 NHTHEDFIDEEQSPVVYHPIWLINTV------GLQ
53. VIT_03s0017g00670 EGAHDDFIDEDQEPVVDHPIWYIRTV------GLQ
54. VIT_18s0001g06640 DGRDEEFLDENRGPAIDHPIWFITTA------GLQ
55. VIT_18s0001g01050 KSRADE--------------------------GLS
56. VIT_18s0001g01060 LGQQPELPAPVKPESEK---------------GLS
57. VIT_02s0012g01440 ENQDINEELEDNHNPALHEPWHVATT------GLD
58. VIT_11s0016g01430 HHFFDPRPSQSFENMNLGLVSSFKYR
59. VIT_09s0002g01500 NTSLGEKSFASR--------------------SLN
60. VIT_13s0019g01020 MVSPGSVNSSD
61. VIT_13s0019g01000 STAAPTGPQQLDRPLTYED-------------GATP
62. VIT_13s0019g00990 QRSDRERLQQLMYQRADQTPSSVDN
63. VIT_14s0083g00710 DYVNATEYVTLFGLFTEDDAGLKGSP------PAA
64. VIT_17s0000g04730 LADHYDYIYTSEYDMLFGQFAENENALT----GRPPAS
65. VIT_19s0015g01000 CCCVPLVSNLLGYNMNMGSVDK----------GAS
66. VIT_09s0002g05130 MAPRNLTVTATADLFIFKAPPTR
67. VIT_09s0002g05140 RCAAFCLLLPFLILPTLASPQEQARE------ANP
68. VIT_11s0016g04450 ILGHGDLSQVR---------------------GAS
69. VIT_04s0008g04280 ILGFREDLTQTR--------------------GAT
70. VIT_09s0002g05120 ILGFREDLSQTR--------------------GAA
71. VIT_11s0016g03190 NRHIDLCFDLDLAITAVDDTSEISKS
72. VIT_13s0074g00370 NASRRSFPVASRSDLSILERGLH---------GLE
73. VIT_15s0048g01840 MFEIDSRIDLEQPEHRIS--------------GLE
74. VIT_08s0056g00320 FGNSEDYLDARGFEEFLEHLAETDSSRR----GAPPAA
75. VIT_06s0004g05080 ALLDREFDDLDYETLRSLDSDNTSST------SLS
76. VIT_13s0019g01960 LLDREFDDLDYETLRALDSENVPTTP------SMS
77. VIT_13s0156g00140 DTNNDGNDSSDLPPPRYLNPNSVAAS
78. VIT_17s0053g00320 DYFIGPGLEQLIQQLAENDPNRYGTP------PAS
79. VIT_08s0007g00720 PASVSEFLMGSGFDRLLDQLAQLEIN------GAGRCEHPPAS
80. VIT_06s0004g05090 MGSGFDRLLEQLSQIEINGFGRGEHP------PAS
81. VIT_13s0019g01980 LGSGFDRLLEQLSQMEINGIGRYEQP------PAS
82. VIT_06s0009g02350 GGNVGDLFLGPGLEELIEQLTMNDRR------GPPPAT
83. VIT_08s0040g00590 RGNVGDYFIGPGLEELFEQLSVNDRR------GPPPAS
84. VIT_18s0001g03270 RFELRDFFSGPGLNELIEELTQNDRP------GPPPAP
85. VIT_12s0034g01390 RDVLNEEMEELTHNEVTGENTQDDRP------GPPPAA
86. VIT_12s0034g01400 RDVLNEAMEELTHNEVTGENTQNDRP------GPPPAA
87. VIT_18s0001g14530 TGNPEDYVDAAGYEELLQNLAESDGAARRGAPPAS
88. VIT_19s0090g00400 QWQISTLETNYGETEDFYNISGAK--------GLP
89. VIT_00s0264g00020 QSQMGAVEVSYDEVPNIFDTGGAK--------GLP
90. VIT_17s0000g06460 NLSYDEMYDVYGEVASR---------------GLS
91. VIT_05s0051g00730 LQIQRDFNENDYEMLLALDENNHNV-------GAS
92. VIT_06s0004g00120 DYFIGPSLDLLLQHLAENDPNRYGTP------PAQ
93. VIT_17s0000g08400 MPS
94. VIT_12s0028g01580 QPHPTSPKCSLQATLEFPDQWHIQMPAHEEQPRLTRAEQ
95. VIT_12s0028g01560 EQSRFTHNEQNKAFKKLRKEFYIPKP
96. VIT_12s0028g01570 TRQASPYQEQSKLTQDEQKVALQKLK

Supplementary Figure 4. Sequence LOGO and alignment of the grapevine ATL GLD domains. Both LOGO and alignment were generated with Geneious (MUSCLE algorithm) using the protein sequences of the GLD domain of the 96 ATLs identified in the whole *Vitis vinifera* genome. The colour index indicates the level of amino acid conservation among the 96 sequences, with dark yellow indicating 80 to 100% of residue conservation, light yellow 60 to 80% of conservation and white a conservation level <60%.

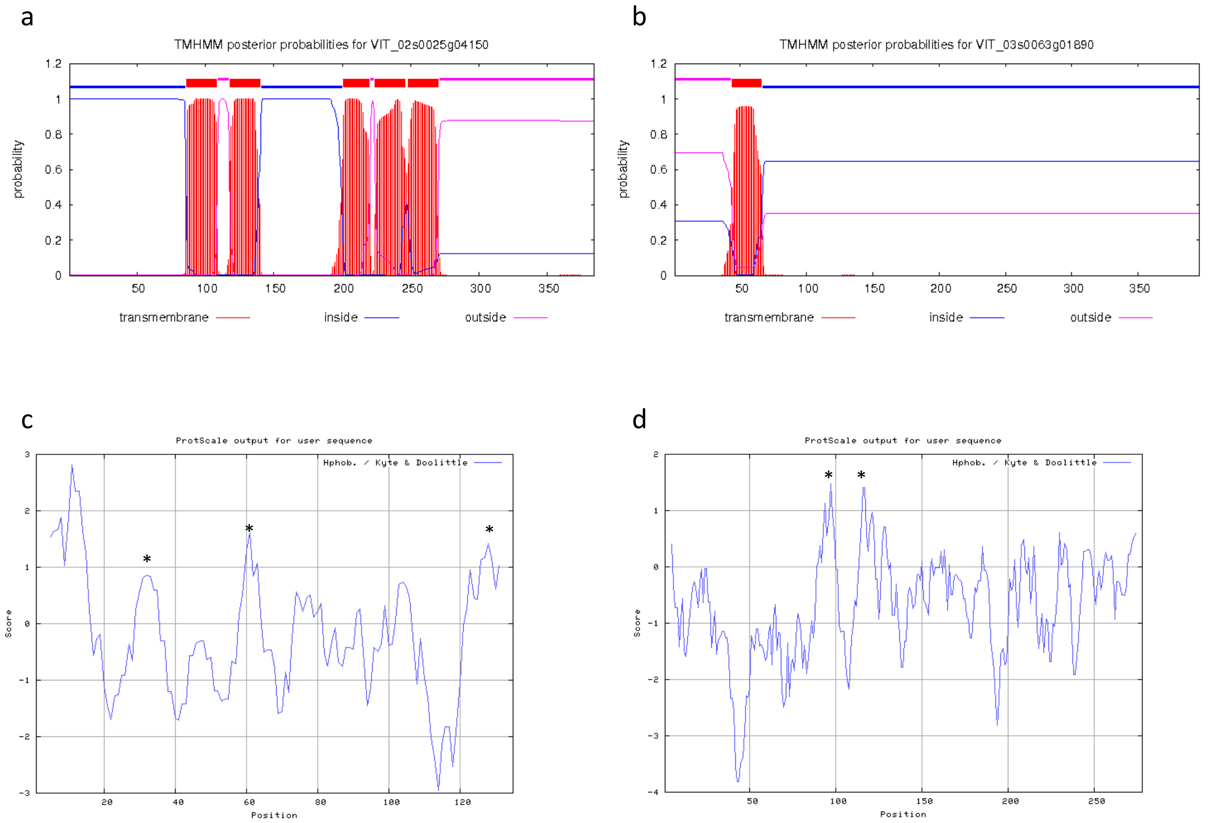

Supplementary Figure 5. Examples of predicted transmembrane and hydrophobic domains in grapevine ATL proteins. a-b, Diagrams were created using TMHMM Server v2.0 (http://www.cbs.dtu.dk/services/TMHMM/). The ordinate displays transmembrane domain probability. The abscissa shows the amino acid number and red bars represent predicted transmembrane domains. c-d, Diagrams were created using the ProtScale tool of Expasy (http://web.expasy.org/protscale/) using the method of Kyte and Doolittle. The ordinate displays hydrophobic and hydrophilic values, plotted above and below 0, respectively. The abscissa shows the amino acid number and the asterisks (*) represent the hydrophobic segments of the protein.

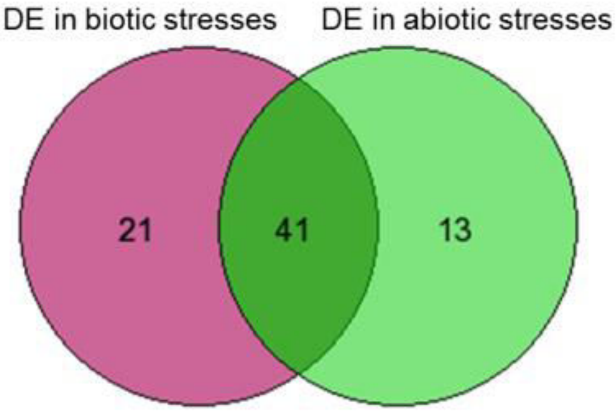

Supplementary Figure 6. Venn diagram showing the number of unique and common differentially expressed ATLs in grapevine in response to biotic (purple) or abiotic (green) stresses. The Venn diagram was generated using VENNY (http://bioinfogp.cnb.csic.es/tools/venny/index.html). DE, differentially expressed.

Supplementary Table 1: Details on ATL gene position in *V. vinifera* genome, duplication state and ATL protein physico-chemical characteristics and location.

[a] number of phosphorylation sites predicted by Musite.
[b] similar predictions obtained with at least two software are highlighted in bold; ngLOC was used with default settings, whereas TargetP v1.1 and Protein Prowler Subcellular Localisation were used with a cut-off of probability of 0.5.

NUC, nucleus; MIT, mitochondria; CHL, chloroplast; PLA, plasma membrane; S, secretory pathway (presence of a signal peptide); M, mitochondria, C, chloroplast; O or - , other locations; nd, not determined (i.e. value below the threshold).

**Name**	**Gene ID**	**Chr.**	**Gene position**		**Strand**	**Duplication state**	**Mol. Wt (kDa)**	**pI**	**P sites[a]**	**Subcellular location [b]**		
			**Start**	**End**						**ngLOC**	**TargetP**	**PProwler**
VviATL3	VIT_09s0002g00220	9	2,E+02	2,E+02	plus	Dispersed	33.08	5.7	1	NUC	**S**	**S**
VviATL4[VviRHX1A]	VIT_15s0021g00890	15	10,761,195	10,763,021	minus	Dispersed	22.12	4.8	1	**NUC**	_	**O**
VviATL18	VIT_11s0118g00780	11	6,552,717	6,553,829	minus	Dispersed	21.59	9.4	0	**MIT**	_	**M**
VviATL23a	VIT_18s0001g01060	18	1,727,361	1,728,295	minus	Tandem	12.33	4.8	11	**MIT**	**M**	O
VviATL23b	VIT_18s0001g01050	18	1,721,216	1,721,614	plus	Tandem	14.79	5	0	CHL	**S**	**S**
VviATL24	VIT_17s0000g06460	17	7,045,832	7,050,297	minus	Dispersed	23.41	5.6	5	NUC	**S**	**S**
VviATL27	VIT_00s0264g00020	Un	18,991,085	18,993,638	minus	Dispersed	25.36	5	0	CHL	_	S
VviATL43	VIT_11s0052g00530	11	17,936,593	17,938,168	minus	Dispersed	51.48	9.6	0	CHL	_	S
VviATL54a	VIT_18s0001g06640	18	5,000,284	5,003,505	plus	WGD	44.98	5.2	5	MIT	**S**	**S**
VviATL54b	VIT_03s0017g00670	3	15,529,867	15,532,640	minus	Dispersed	47.50	5.4	1	NUC	S	C
VviATL55[VviRING1]	VIT_07s0191g00230	7	15,035,569	15,037,412	plus	WGD	41.30	6	3	NUC	C	S
VviATL63	VIT_06s0004g06930	6	7,643,002	7,643,805	plus	Dispersed	29.33	5.8	0	NUC	_	S
VviATL65	VIT_03s0063g01890	3	5,217,040	5,219,107	minus	Dispersed	45.16	10	1	NUC	M	nd
VviATL82	VIT_01s0026g02540	1	12,168,327	12,169,146	plus	Dispersed	25.68	4.9	4	PLA	_	O
VviATL83	VIT_17s0000g08400	17	9,599,237	9,601,123	plus	Dispersed	16.05	6.1	2	**NUC**	C	**O**
VviATL84	VIT_06s0004g00120	6	3,E+02	3,E+02	plus	Dispersed	40.90	4.8	1	CHL	_	O
VviATL85	VIT_12s0034g01400	12	17,414,404	17,415,189	plus	Tandem	29.71	6.9	2	**CHL**	**C**	S
VviATL86	VIT_12s0034g01390	12	17,398,801	17,400,234	plus	Tandem	52.26	8.2	1	CHL	**M**	**M**
VviATL87	VIT_18s0001g03270	18	3,233,803	3,234,804	minus	Dispersed	38.18	7.1	7	PLA	_	M
VviATL88	VIT_08s0040g00590	8	11,540,810	11,542,129	plus	Dispersed	34.85	7.7	0	NUC	_	-
VviATL89	VIT_06s0009g02350	6	14,742,953	14,747,814	minus	Dispersed	36.99	6.3	4	CHL	C	S

VviATL90	VIT_06s0004g05090	6	6,020,330	6,022,057	minus	WGD	42.57	8.9	3	CHL	_	S
VviATL91	VIT_13s0019g01980	13	3,282,264	3,294,013	minus	WGD	83.58	7.8	0	CHL	_	O
VviATL92	VIT_08s0007g00720	8	14,916,670	14,922,764	minus	WGD	57.58	8.6	3	NUC	**C**	**C**
VviATL93	VIT_13s0156g00140	13	23,884,150	23,887,948	plus	Dispersed	33.41	4.7	8	CHL	M	S
VviATL94	VIT_17s0053g00320	17	14,868,182	14,869,346	minus	Dispersed	38.60	4.4	6	MIT	**S**	**S**
VviATL95	VIT_04s0008g02290	4	1,889,354	1,891,807	minus	Proximal	32.81	5	3	NUC	_	nd
VviATL96	VIT_08s0056g00320	8	5,E+02	5,E+02	minus	Dispersed	66.55	4.6	2	NUC	_	M
VviATL97	VIT_11s0016g03190	11	2,563,204	2,564,128	plus	Dispersed	17.85	6.4	1	CHL	_	S
VviATL98	VIT_17s0000g04730	17	5,126,163	5,127,854	minus	WGD	49.33	4.1	1	**PLA**	_	**O**
VviATL99	VIT_14s0083g00710	14	22,855,118	22,856,402	minus	WGD	43.71	4	0	CHL	_	S
VviATL100	VIT_08s0040g02950	8	13,968,494	13,969,465	plus	Dispersed	31.37	7.6	16	NUC	**M**	**M**
VviATL101	VIT_18s0001g14530	18	12,533,609	12,536,412	plus	Dispersed	37.20	4.3	1	**NUC**	_	**O**
VviATL102	VIT_02s0012g01440	2	7,633,461	7,635,180	plus	Dispersed	33.14	5	5	NUC	**S**	**S**
VviATL103	VIT_19s0090g00400	19	6,551,526	6,553,119	minus	Dispersed	24.03	6.5	0	CHL	**M**	**M**
VviATL104	VIT_05s0051g00730	5	11,700,888	11,720,325	minus	Dispersed	21.26	6.1	8	NUC	_	S
VviATL105	VIT_13s0064g01030	13	22,907,333	22,911,899	minus	WGD	28.32	5.5	1	NUC	_	M
VviATL106	VIT_06s0004g01930	6	2,373,671	2,382,922	plus	WGD	28.69	5.5	4	END	_	S
VviATL107	VIT_18s0075g00220	18	21,471,547	21,496,753	minus	Dispersed	47.91	8.9	1	NUC	**S**	**S**
VviATL108	VIT_04s0023g03580	4	20,118,656	20,123,142	plus	Tandem	46.61	6.1	8	PLA	M	S
VviATL109	VIT_11s0052g00360	11	17,683,751	17,684,146	minus	Dispersed	14.89	4.8	0	CHL	**S**	**S**
VviATL110	VIT_11s0118g00760	11	6,533,770	6,542,103	plus	Dispersed	58.69	5.4	0	NUC	**M**	**M**
VviATL111	VIT_02s0087g00420	2	17,748,161	17,758,528	plus	Dispersed	62.51	8.2	5	CHL	_	C
VviATL112	VIT_19s0015g01000	19	9,081,946	9,084,990	plus	Dispersed	39.31	8.8	0	NUC	**C**	**C**
VviATL113	VIT_11s0016g04450	11	3,757,897	3,762,458	minus	WGD	44.19	5.5	6	PLA	_	S
VviATL114	VIT_04s0008g04280	4	3,660,980	3,669,030	plus	WGD	44.19	7.9	0	CHL	_	O
VviATL115	VIT_09s0002g05120	9	4,813,804	4,820,015	minus	WGD	48.81	6.5	1	CYT	**S**	**S**
VviATL116	VIT_09s0002g05140	9	4,836,806	4,838,491	minus	Tandem	33.91	5.2	1	PLA	**S**	**S**
VviATL117	VIT_09s0002g05130	9	4,821,806	4,822,755	minus	Tandem	22.69	5.8	1	NUC	_	S
VviATL118	VIT_12s0057g01330	12	10,069,923	10,070,893	minus	Dispersed	22.49	5.9	2	CHL	**S**	**S**
VviATL119	VIT_08s0040g00310	8	11,231,370	11,255,659	minus	Dispersed	43.10	6.8	1	CYT	**M**	**M**
VviATL120	VIT_13s0019g01960	13	3,261,036	3,269,087	minus	WGD	30.08	6.2	5	CHL	**M**	**M**
VviATL121	VIT_06s0004g05080	6	6,014,664	6,018,771	minus	WGD	31.83	5.7	7	NUC	**M**	**M**
VviATL122	VIT_17s0000g08210	17	9,272,873	9,280,391	plus	Dispersed	49.13	6	1	CHL	_	O
VviATL123	VIT_03s0091g00480	3	6,851,584	6,855,075	minus	Dispersed	31.25	8.6	1	NUC/CYT	_	M
VviATL124	VIT_04s0023g03460	4	20,022,053	20,022,628	plus	Dispersed	18.05	7	0	CHL	S	nd
VviATL125	VIT_00s0349g00040	Un	24,940,977	24,941,538	plus	Dispersed	15.22	8.1	0	CHL	S	S

VviATL126	VIT_12s0028g01580	12	2,278,915	2,280,538	plus	Tandem	38.82	6.6	4	NUC	_	O
VviATL127	VIT_12s0028g01560	12	2,265,770	2,266,849	plus	Tandem	22.29	8.8	0	NUC	**M**	**M**
VviATL128	VIT_12s0028g01570	12	2,274,491	2,275,931	plus	Tandem	26.20	7.6	0	CYT	**M**	**M**
VviATL129	VIT_15s0048g02030	15	16,165,343	16,170,176	plus	Dispersed	43.09	5.7	0	CHL	**S**	**S**
VviATL130	VIT_02s0025g04150	2	3,670,653	3,678,402	plus	Dispersed	42.55	5.2	5	**CHL**	**C**	nd
VviATL131	VIT_15s0048g01840	15	15,988,642	15,991,207	minus	Dispersed	22.58	5.5	5	NUC	**C**	**C**
VviATL132	VIT_13s0074g00370	13	7,973,641	7,981,844	plus	Dispersed	23.54	5.8	1	CHL	_	S
VviATL133	VIT_18s0089g00860	18	28,686,085	28,689,586	plus	Dispersed	24.97	5.1	1	NUC	_	S
VviATL134	VIT_06s0004g08080	6	8,843,648	8,845,413	plus	WGD	29.12	8	1	NUC	**M**	**M**
VviATL135	VIT_08s0058g01270	8	10,736,680	10,737,686	plus	WGD	21.19	6.9	9	NUC	_	S
VviATL136	VIT_15s0024g01990	15	4,356,512	4,356,925	plus	Dispersed	14.73	5.9	2	PLA	_	S
VviATL137	VIT_13s0067g02880	13	1,558,971	1,559,978	minus	Dispersed	21.06	6.7	0	PLA	**M**	**M**
VviATL138	VIT_03s0038g03930	3	2,873,799	2,874,340	plus	WGD	14.05	8.9	10	NUC	_	-
VviATL139	VIT_18s0122g00870	18	6,E+02	6,E+02	minus	WGD	19.24	8.3	0	CHL	_	S
VviATL140	VIT_07s0031g02250	7	18,383,520	18,384,391	plus	WGD	19.29	8.3	3	PLA	C	M
VviATL141	VIT_11s0065g01210	11	15,329,284	15,329,979	minus	Dispersed	18.78	8.8	8	CHL	**M**	**M**
VviATL142	VIT_15s0046g02070	15	18,873,629	18,874,670	minus	Dispersed	21.47	6.5	0	NUC	**S**	**S**
VviATL143	VIT_13s0084g00140	13	18,797,445	18,798,197	minus	Dispersed	20.08	7.6	2	CHL	C	O
VviATL144	VIT_02s0025g01430	2	1,379,376	1,383,064	minus	WGD	42.37	6.1	4	NUC	_	S
VviATL145	VIT_16s0098g00250	16	20,642,143	20,644,650	minus	Dispersed	40.74	5.5	0	**CYT**	_	**O**
VviATL146	VIT_15s0046g00930	15	17,988,199	17,990,082	plus	WGD	40.71	4.8	2	NUC	C	S
VviATL147	VIT_18s0001g06670	18	5,020,431	5,021,751	plus	Dispersed	44.25	9.1	2	MIT	**S**	**S**
VviATL148	VIT_14s0128g00120	14	2,814,506	2,816,124	plus	Dispersed	45.76	9.5	5	CHL	_	O
VviATL149	VIT_12s0028g02530	12	3,292,336	3,293,351	plus	WGD	27.54	5.7	3	CYT	**M**	**M**
VviATL150	VIT_10s0003g00850	10	2,100,756	2,101,775	minus	WGD	23.74	5.2	1	CHL	_	O
VviATL151	VIT_11s0016g03420	11	2,778,391	2,787,457	plus	Dispersed	53.37	7.2	6	CHL	_	O
VviATL152	VIT_11s0037g01400	11	10,944,676	10,946,544	minus	Dispersed	59.50	6.3	0	CHL	_	O
VviATL153	VIT_01s0026g00300	1	8,988,327	8,989,738	plus	WGD	45.76	6	6	**CHL**	**C**	**C**
VviATL154	VIT_14s0066g01610	14	27,970,245	27,971,983	minus	WGD	42.89	6.2	1	**NUC**	_	**O**
VviATL155	VIT_07s0005g00710	7	3,351,600	3,352,612	plus	WGD	28.82	6.7	1	PLA	_	S
VviATL156	VIT_05s0077g01970	5	1,538,565	1,539,938	minus	WGD	34.99	8.3	8	NUC	_	O
VviATL157	VIT_13s0019g01020	13	2,749,369	2,749,848	plus	Tandem	17.49	6.8	1	END	_	S
VviATL158	VIT_13s0019g01000	13	2,742,040	2,742,492	plus	Tandem	16.69	4.6	1	NUC	**S**	**S**
VviATL159	VIT_13s0019g00990	13	2,740,552	2,741,055	plus	Tandem	18.34	5.9	0	CHL	**S**	**S**
VviATL160	VIT_09s0002g01500	9	1,282,580	1,283,385	plus	WGD	15.83	4.9	1	**NUC**	_	**O**
VviATL161	VIT_11s0016g01430	11	1,146,136	1,146,786	plus	WGD	20.16	6.4	0	**CHL**	**C**	O
VviATL162	VIT_07s0005g03120	7	5,941,893	5,942,809	minus	WGD	29.97	6.1	0	CHL	_	S

VviATL163	VIT_05s0049g00480	5	7,527,982	7,529,676	minus	WGD	43.91	8.3	0	NUC	**C**	**C**
VviATL164	VIT_15s0045g00330	15	5,087,898	5,088,931	plus	Dispersed	37.58	5.6	1	PLA	**S**	**S**

Supplementary Table 4: List of grapevine ATLs differentially expressed in response to biotic and abiotic stresses and responsiveness specificity.

Name	Gene ID	Stress
VviATL18	VIT_11s0118g00780	biotic and abiotic
VviATL23a	VIT_18s0001g01060	biotic and abiotic
VviATL24	VIT_17s0000g06460	biotic and abiotic
VviATL27	VIT_00s0264g00020	biotic and abiotic
VviATL3	VIT_09s0002g00220	biotic and abiotic
VviATL4[VviRHX1A]	VIT_15s0021g00890	abiotic
VviATL43	VIT_11s0052g00530	abiotic
VviATL54a	VIT_18s0001g06640	abiotic
VviATL54b	VIT_03s0017g00670	biotic and abiotic
VviATL55[VviRING1]	VIT_07s0191g00230	biotic
VviATL65	VIT_03s0063g01890	biotic and abiotic
VviATL82	VIT_01s0026g02540	biotic
VviATL84	VIT_06s0004g00120	biotic
VviATL88	VIT_08s0040g00590	abiotic
VviATL89	VIT_06s0009g02350	biotic
VviATL90	VIT_06s0004g05090	biotic and abiotic
VviATL91	VIT_13s0019g01980	biotic and abiotic
VviATL92	VIT_08s0007g00720	biotic
VviATL93	VIT_13s0156g00140	biotic
VviATL94	VIT_17s0053g00320	abiotic
VviATL95	VIT_04s0008g02290	biotic
VviATL97	VIT_11s0016g03190	biotic and abiotic
VviATL98	VIT_17s0000g04730	biotic
VviATL99	VIT_14s0083g00710	biotic
VviATL100	VIT_08s0040g02950	biotic and abiotic
VviATL101	VIT_18s0001g14530	biotic
VviATL102	VIT_02s0012g01440	biotic and abiotic
VviATL103	VIT_19s0090g00400	biotic and abiotic
VviATL104	VIT_05s0051g00730	biotic
VviATL105	VIT_13s0064g01030	abiotic
VviATL106	VIT_06s0004g01930	biotic and abiotic
VviATL107	VIT_18s0075g00220	abiotic
VviATL108	VIT_04s0023g03580	biotic
VviATL110	VIT_11s0118g00760	biotic
VviATL111	VIT_02s0087g00420	biotic and abiotic
VviATL112	VIT_19s0015g01000	biotic and abiotic
VviATL113	VIT_11s0016g04450	abiotic
VviATL115	VIT_09s0002g05120	abiotic
VviATL118	VIT_12s0057g01330	biotic and abiotic

VviATL119	VIT_08s0040g00310	biotic and abiotic
VviATL121	VIT_06s0004g05080	biotic and abiotic
VviATL122	VIT_17s0000g08210	biotic and abiotic
VviATL123	VIT_03s0091g00480	biotic
VviATL124	VIT_04s0023g03460	biotic
VviATL125	VIT_00s0349g00040	abiotic
VviATL126	VIT_12s0028g01580	biotic
VviATL127	VIT_12s0028g01560	abiotic
VviATL128	VIT_12s0028g01570	biotic and abiotic
VviATL130	VIT_02s0025g04150	biotic
VviATL132	VIT_13s0074g00370	biotic
VviATL133	VIT_18s0089g00860	biotic and abiotic
VviATL134	VIT_06s0004g08080	biotic and abiotic
VviATL135	VIT_08s0058g01270	biotic and abiotic
VviATL137	VIT_13s0067g02880	biotic and abiotic
VviATL139	VIT_18s0122g00870	biotic and abiotic
VviATL140	VIT_07s0031g02250	biotic and abiotic
VviATL141	VIT_11s0065g01210	biotic and abiotic
VviATL142	VIT_15s0046g02070	biotic and abiotic
VviATL143	VIT_13s0084g00140	biotic and abiotic
VviATL144	VIT_02s0025g01430	biotic and abiotic
VviATL145	VIT_16s0098g00250	biotic
VviATL147	VIT_18s0001g06670	biotic and abiotic
VviATL148	VIT_14s0128g00120	biotic and abiotic
VviATL149	VIT_12s0028g02530	biotic and abiotic
VviATL151	VIT_11s0016g03420	biotic and abiotic
VviATL152	VIT_11s0037g01400	biotic and abiotic
VviATL153	VIT_01s0026g00300	biotic and abiotic
VviATL154	VIT_14s0066g01610	biotic
VviATL155	VIT_07s0005g00710	biotic
VviATL156	VIT_05s0077g01970	biotic and abiotic
VviATL160	VIT_09s0002g01500	abiotic
VviATL161	VIT_11s0016g01430	biotic and abiotic
VviATL162	VIT_07s0005g03120	abiotic
VviATL163	VIT_05s0049g00480	biotic and abiotic
VviATL164	VIT_15s0045g00330	biotic and abiotic
